# Clinical pharmacokinetic properties of magnesium sulphate in women with pre‐eclampsia and eclampsia

**DOI:** 10.1111/1471-0528.13753

**Published:** 2015-11-24

**Authors:** BO Okusanya, OT Oladapo, Q Long, P Lumbiganon, G Carroli, Z Qureshi, L Duley, JP Souza, AM Gülmezoglu

**Affiliations:** ^1^ Experimental and Maternal Medicine (EMM) UnitDepartment of Obstetrics and GynaecologyCollege of MedicineUniversity of LagosIdi‐ArabaLagosNigeria; ^2^ UNDP/UNFPA/UNICEF/WHO/World Bank Special Programme of ResearchDevelopment and Research Training in Human Reproduction (HRP)Department of Reproductive Health and ResearchWorld Health OrganizationGenevaSwitzerland; ^3^ Department of Obstetrics and GynaecologyFaculty of MedicineKhon Kaen UniversityKhon KaenThailand; ^4^ Centro Rosarino de Estudios PerinatalesRosarioArgentina; ^5^ Department of Obstetrics and GynaecologySchool of MedicineCollege of Health SciencesUniversity of NairobiNairobiKenya; ^6^ Nottingham Clinical Trials UnitQueens Medical CentreNottinghamUK; ^7^ Department of Social MedicineRibeirao Preto School of MedicineUniversity of Sao PauloRibeirao PretoSão PauloBrazil

**Keywords:** Eclampsia, magnesium sulphate, pharmacokinetics, pre‐eclampsia, serum magnesium

## Abstract

**Background:**

The pharmacokinetic basis of magnesium sulphate (MgSO_4_) dosing regimens for eclampsia prophylaxis and treatment is not clearly established.

**Objectives:**

To review available data on clinical pharmacokinetic properties of MgSO_4_ when used for women with pre‐eclampsia and/or eclampsia.

**Search strategy:**

MEDLINE, EMBASE, CINAHL, POPLINE, Global Health Library and reference lists of eligible studies.

**Selection criteria:**

All study types investigating pharmacokinetic properties of MgSO_4_ in women with pre‐eclampsia and/or eclampsia.

**Data collection and analysis:**

Two authors extracted data on basic pharmacokinetic parameters reflecting the different aspects of absorption, bioavailability, distribution and excretion of MgSO_4_ according to identified dosing regimens.

**Main results:**

Twenty‐eight studies investigating pharmacokinetic properties of 17 MgSO_4_ regimens met our inclusion criteria. Most women (91.5%) in the studies had pre‐eclampsia. Baseline serum magnesium concentrations were consistently <1 mmol/l across studies. Intravenous loading dose between 4 and 6 g was associated with a doubling of this baseline concentration half an hour after injection. Maintenance infusion of 1 g/hour consistently produced concentrations well below 2 mmol/l, whereas maintenance infusion at 2 g/hour and the Pritchard intramuscular regimen had higher but inconsistent probability of producing concentrations between 2 and 3 mmol/l. Volume of distribution of magnesium varied (13.65–49.00 l) but the plasma clearance was fairly similar (4.28–5.00 l/hour) across populations.

**Conclusion:**

The profiles of Zuspan and Pritchard regimens indicate that the minimum effective serum magnesium concentration for eclampsia prophylaxis is lower than the generally accepted level. Exposure–response studies to identify effective alternative dosing regimens should target concentrations achievable by these standard regimens.

**Tweetable abstract:**

Minimum effective serum magnesium concentration for eclampsia prophylaxis is lower than the generally accepted therapeutic level.

## Tweetable abstract

Minimum effective serum magnesium concentration for eclampsia prophylaxis is lower than the generally accepted therapeutic level.

## Introduction

Magnesium sulphate (MgSO_4_) has been used to treat pre‐eclampsia and eclampsia for more than a century and is currently the anticonvulsant of choice for the prevention and control of eclamptic fits.^1^, ^2^ Historically, the total dose of MgSO_4_ used for treating pre‐eclampsia and eclampsia was gradually increased from as low as 2 g/24 hours to as high as 54 g/24 hours with the belief that this would increase clinical efficacy.^3^, ^4^, ^5^ All these studies reported good control of convulsions despite the considerable variations in the regimen, route of administration and total dose of MgSO_4_ used. The mechanism of action of MgSO_4_ in eclampsia prophylaxis and treatment remains poorly understood and to date, there has been no rigorous evaluation of therapeutic serum magnesium concentration for preventing or treating eclamptic seizures. The so‐called minimum therapeutic level of 2 mmol/l has been suggested based on clinical and laboratory observations in earlier studies rather than standard exposure–response studies.^3^, ^6^

The lack of knowledge on how MgSO_4_ works is supported by reports of clinical efficacy among pre‐eclamptic women with lower serum magnesium levels, and clinical failure among those with serum magnesium levels within the generally accepted therapeutic range.^7^, ^8^ The two currently recommended regimens (Zuspan and Pritchard) have been internationally accepted as standard regimens on the basis of their proven clinical efficacy in the two largest MgSO_4_ trials.^1^, ^2^ Although these trials showed comparable clinical efficacy for the predominantly intramuscular (Pritchard) and intravenous (Zuspan) regimens, they also highlighted the lack of understanding of the minimum effective dose for eclampsia prevention and treatment.

More recently, concerns about adverse events with the use of standard regimens, and coverage limitations posed by health resource requirements in low‐income settings^9^ have renewed interest in identifying the minimum effective dose of MgSO_4_ for preventing and treating eclampsia. In response, WHO has embarked on a research project to identify a simpler MgSO_4_ regimen based on the minimum dose required to achieve clinical efficacy. An initial step of this effort requires a comprehensive review of pharmacokinetic data that are available for MgSO_4_, which along with the related efficacy data will help to establish the serum magnesium levels that should be targeted in standard exposure–response studies. The aim of this study was to systematically review available data on clinical pharmacokinetic properties of MgSO_4_ when used for the treatment of pre‐eclampsia and eclampsia.

## Methods

We prepared this review in accordance with PRISMA guidelines and followed a protocol. Eligible studies included observational and experimental studies where MgSO4 was used for eclampsia prophylaxis and/or treatment, and a complete or partial pharmacokinetic profile of MgSO_4_ was reported, irrespective of the routes and duration of administration or dosage regimen. Participants were women who received MgSO_4_ for the prevention or treatment of eclampsia, regardless of their gestational age at treatment or pregnancy outcomes. We did not impose any restrictions based on the number of participants involved in the study. For the purpose of this review, where a study had another arm of women who were not pregnant, had a normal pregnancy, or received MgSO_4_ for reasons other than pre‐eclampsia and eclampsia, we only extracted data for women with pre‐eclampsia and eclampsia. Where such data disaggregation and extraction were impossible, we excluded the study from the review.

The outcomes of interest consisted of basic pharmacokinetic parameters reflecting the different aspects of absorption, bioavailability, distribution and excretion according to the various dosing regimens identified. These included baseline, peak and steady‐state serum magnesium concentrations through the period of drug administration, percentage ionised magnesium (Mg^2+^), volume of distribution, central nervous system and fetal distribution, plasma renal clearance, half‐life, and serum concentrations associated with toxic side effects.

We searched MEDLINE, CINAHL, POPLINE, and Global Health Library in October and November 2013 and updated the search in March 2015. The detailed search strategies are included in Table 1. We also searched the reference lists of all eligible studies. No language or date restrictions were applied.

**Table 1:** Search strategies

Electronic databases				
MEDLINE	POPLINE	GLOBAL HEALTH LIBRARY	CINAHL	EMBASE
‘Magnesium Sulfate’[Mesh] OR ‘Magnesium Sulfate’ OR ‘Magnesium Sulphate’ OR ‘MgSO4′ OR ‘7487‐88‐9’ [RN] OR ‘mg longoral’ OR ‘sulfamag’ OR sulmetin OR sulmetine AND ‘Hypertension, Pregnancy‐Induced’[Mesh] OR ‘Pregnancy Toxemias’[Mesh] OR ‘pre‐eclampsia’ OR ‘preeclampsia’ OR ‘pregnancy toxemia’ OR ‘pregnancy toxemias’ OR ‘eclampsia’ OR ‘eclampsias’	(magnesium sulfate) OR (magnesium sulphate)	[(magnesium sulfate) OR (magnesium sulphate)] AND [(pre‐eclampsia) OR (preeclampsia) OR (eclampsia) OR (eclampsias)]	TX ‘gestational hypertension’ OR TX ‘hypertension in pregnancy’ OR TX ‘maternal hypertension’ OR TX ‘pregnancy hypertension’ OR TX ‘pregnancy induced hypertension’ OR TX ‘maternal hypertension’ OR TX ‘pregnancy toxemia’ OR TX ‘eclamptic toxemia’ OR TX ‘eclamptogenic toxemia’ OR TX ‘EPH gestosis’ OR TX ‘eph syndrome’ OR TX ‘gestational toxemia’ OR TX ‘gestational toxicosis’ OR TX ‘gestosis’ OR TX ‘gestosis, eph’ OR TX ‘hep syndrome’ OR TX ‘pregnancy toxaemia’ OR TX ‘pregnancy toxemias’ OR TX ‘pregnancy toxicosis’ OR TX ‘toxemia gravidum’ OR TX ‘toxemic pregnancy’ OR TX ‘toxicosis gravidarum’ OR TX ‘pre eclampsia’ OR TX ‘pre eclamptic toxaemia’ OR TX ‘pre eclamptic toxemia’ OR TX ‘preclampsia’ OR TX ‘preeclamptic toxaemia’ OR TX ‘preeclamptic toxemia’ OR TX ‘puerperal tetany’ OR TX ‘eclampsia’ OR TX ‘eclampsias’ OR (MH ‘Pregnancy‐Induced Hypertension+’) OR (MH ‘Eclampsia+’) OR (MH ‘Pre‐Eclampsia+’) AND (MH ‘Magnesium Sulfate’) OR TX ‘magnesium sulfate’ OR TX ‘magnesium sulfate’ OR TX MgSO4 OR TX ‘7487‐88‐9’ OR TX ‘mg longoral’ OR TX ‘sulfamag’ OR TX sulmetin OR TX sulmetine OR TX ‘Magnesium sulphate’	‘gestational hypertension’ OR ‘hypertension in pregnancy’ OR ‘maternal hypertension’ OR ‘pregnancy hypertension’ OR ‘pregnancy induced hypertension’ OR ‘maternal hypertension’/exp OR ‘pregnancy toxemia’/exp OR ‘eclamptic toxemia’ OR ‘eclamptogenic toxemia’ OR ‘EPH gestosis’ OR ‘eph syndrome’ OR ‘gestational toxemia’ OR ‘gestational toxicosis’ OR ‘gestosis’ OR ‘gestosis, eph’ OR ‘hep syndrome’ OR ‘pregnancy toxaemia’ OR ‘pregnancy toxemias’ OR ‘pregnancy toxicosis’ OR ‘toxemia gravidum’ OR ‘toxemic pregnancy’ OR ‘toxicosis gravidarum’ OR ‘eclampsia and preeclampsia’/exp OR ‘pre‐eclampsia’ OR ‘pre‐eclamptic toxaemia’ OR ‘pre‐eclamptic toxemia’ OR ‘pre eclampsia’ OR ‘preclampsia’ OR ‘preeclamptic toxaemia’ OR ‘preeclamptic toxemia’ OR ‘puerperal tetany’ OR ‘eclampsia’ OR ‘eclampsias'AND ‘magnesium sulfate’/exp OR ‘magnesium sulfate’ OR mgso4 OR ‘7487‐88‐9’:rn OR ‘mg longoral’ OR ‘sulfamag’ OR sulmetin OR sulmetine OR ‘magnesium sulfate’/de OR ‘Magnesium sulphate'AND NOT [‘animal’/exp NOT (‘animal’/exp AND ‘human’/exp)]

BOO and OTO independently assessed the initial search outputs for potentially eligible studies. BOO and QL assessed the search outputs in the updated search. BOO and OTO independently extracted data using a standardised data form. Any discrepancies were resolved through discussion and consensus between the two authors. We included data from studies that combined women with pre‐eclampsia and eclampsia where it was impossible to disaggregate the data accordingly. Where possible, we separately extracted data for women with pre‐eclampsia and eclampsia.

Given the significant methodological heterogeneity, variations in reporting format and sparseness of pharmacokinetic data among studies reporting on the same dosage regimen, the available pharmacokinetic parameters for each MgSO_4_ regimen identified were qualitatively synthesised according to the two predominant routes of administration—intravenous and intramuscular. Findings were presented as continuous data with measures of central tendency and distribution as reported by the original authors.

For the purpose of this review, we expressed all magnesium levels in the standard international (SI) units [millimole/litre (mmol/l)] by applying standard conversion factors as appropriate. To convert to mmol/l, the reported serum magnesium values in milligram/decilitre (mg/dl) and milliequivalent/litre (mEq/l) were multiplied by 0.411 and 0.500, respectively.^10^

We designed a checklist and criteria for quality assessment based on a modification of the QUADAS‐2 tool^11^ (Table S1). The checklist has eight domains: adequacy of sample size for reliable pharmacokinetic study; representativeness of involved participants to population of interest; adequate reporting of co‐variates; study primary objective; reporting of details of laboratory methods used; relevance of laboratory method to contemporary practice; reporting of baseline magnesium level; and duration of follow‐up and attrition bias. We considered the overall risk of bias of a study to be ‘low’ when four or more of the above listed domains were assessed to be at low risk of bias; ‘uncertain’ when the risk of bias was unclear in five or more domains, or in four domains but with high risk of bias in any of the remaining domains; and ‘high’ when the risk of bias was assessed as high in three or more domains.

## Results

**Figure 1:**
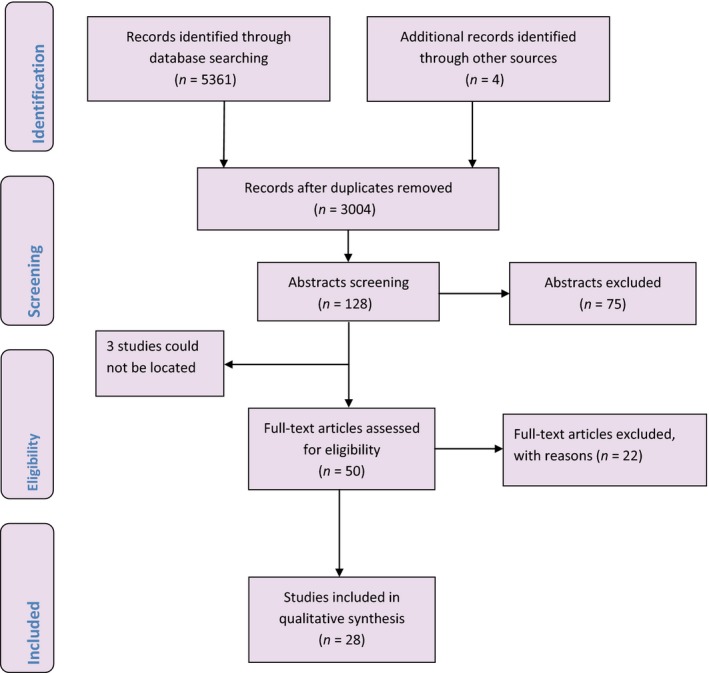
Detailed data selection process.

The search strategies yielded 5361 citations from the electronic databases and four additional citations from other sources. Fifty‐three potentially eligible studies were identified after screening of titles and abstracts and removal of duplicates (Figure 1). Full texts of 50 of these 53 studies (three could not be located) were retrieved and assessed. Twenty‐eight studies published over a span of seven decades and conducted in the Americas, Europe, Asia, and sub‐Saharan Africa met the inclusion criteria.^3^, ^5^, ^8^, ^12^, ^13^, ^14^, ^15^, ^16^, ^17^, ^18^, ^19^, ^20^, ^21^, ^22^, ^23^, ^24^, ^25^, ^26^, ^27^, ^28^, ^29^, ^30^, ^31^, ^32^, ^33^, ^34^, ^35^, ^36^ The studies involved a total of 1466 women with pre‐eclampsia and/or eclampsia. The majority (91.5%) of these women received MgSO_4_ for the treatment of pre‐eclampsia and a smaller proportion (8.5%) received the drug for treatment of eclampsia. The study designs were cross‐sectional, case‐control, randomised and non‐randomised trials. Four of the studies used model‐based techniques to determine pharmacokinetic parameters (see characteristics of included studies in Table S2). Twenty‐two studies were excluded from this review for various reasons (see list of excluded studies in Box S1 and their characteristics in Table S3). Overall, the included studies were very heterogeneous mainly in relation to how blood samples were collected, the timing of serum magnesium estimation, storage method prior to laboratory investigation and laboratory techniques used to estimate magnesium levels.

Table S4 and Figures S1–S3 present the risk of bias assessment for all domains across the included studies, according to the various intravenous and intramuscular regimens identified. Overall, 12 studies were assessed to be at low risk of bias,^5^, ^8^, ^17^, ^18^, ^19^, ^22^, ^26^, ^28^, ^29^, ^32^, ^33^, ^35^ 13 at uncertain risk of bias^3^, ^12^, ^13^, ^14^, ^15^, ^16^, ^20^, ^24^, ^25^, ^27^, ^30^, ^34^, ^36^ and 3 at high risk of bias.^21^, ^23^, ^31^ For 23 studies that examined intravenous regimens, 10 were assessed to be at low risk of bias^5^, ^8^, ^17^, ^18^, ^19^, ^22^, ^26^, ^32^, ^33^, ^35^, 11 at uncertain risk of bias^3^, ^12^, ^13^, ^14^, ^16^, ^24^, ^25^, ^27^, ^30^, ^34^, ^36^ and two at high risk of bias.^21^, ^23^ For the nine studies that examined only intramuscular regimens, three studies were assessed to be at low risk of bias^5^, ^28^, ^29^ five at uncertain risk of bias^3^, ^15^, ^16^, ^20^, ^30^ and one at high risk of bias.^31^

### Intravenous regimens

#### 4‐g loading dose and 1 g/hour continuous maintenance infusion (Zuspan regimen)

Seven studies reported pharmacokinetic data based on the Zuspan regimen.^5^, ^12^, ^17^, ^18^, ^24^, ^27^, ^33^ The reported baseline levels were all <1.00 mmol/l with mean values ranging between 0.74 and 0.85 mmol/l (Table 2). Following the loading dose, serum magnesium levels rose sharply to about twice the baseline levels at ½ hour (1.48–1.70 mmol/l). At 1, 2, and 4 hours of the maintenance dose, the mean serum levels remained at a fairly constant level that was consistent with the values attained at ½ hour. The serum levels at 8, 12, and 24 hours also remained within the same range and at no point did the mean serum concentration level reached 2.00 mmol/l. The described serum magnesium levels between ½ hour and 24 hours following initiation of treatment was consistent with the steady‐state level of 1.64 mmol/l and an ‘average concentration’ of 1.70 mmol/l reported by two studies.^5^, ^17^ One study showed that the peak serum concentration was achieved within half an hour of treatment.^24^ The apparent volume of distribution estimated by two model‐based studies varied considerably between the populations studied—15.60 l in a population of Indian women compared with 32.20 l in Australian women.^17^, ^27^ However, the estimated plasma clearance was fairly consistent across the two populations—4.81 and 4.28 l/hour, respectively. One study estimated the half‐life of MgSO_4_ to be 5.2 hours.^17^ No other pharmacokinetic parameters were reported in the included studies.

**Table 2:** Serum magnesium concentration‐time profile for intravenous regimens

Regimen	Study	*n*	Mean serum magnesium in mmol/l (SD)								
			Baseline	½ hour	1 hour	2 hours	4 hours	8 hours	12 hours	24 hours	Steady state
4‐g loading + 1 g/hours continuous maintenance	Abbade et al.^12^, ^e^	15	0.74 (0.04)	1.48 (0.16)	1.44 (0.16)	1.36 (0.21)	1.36 (0.21)	–	–	–	–
	Cruikshank et al.^18^, ^d^	20	0.74 (0.03)	–	–	–	–	–	–	1.70^e^	–
	Manorot et al.^24^, ^d^	25	–	1.70 (0.25)	1.62 (0.30)	1.60 (0.23)	1.55 (0.15)	–	–	–	–
	Sibai et al.^5^, ^d^	7	–	–	1.31	1.31	1.44	1.48	1.56	–	1.64
	Tongsong et al.^33^, ^e^	24	–	–	–	1.75	1.76	–	–	–	–
	Chuan et al.^17^, ^a^^,^ e	116	0.81	–	–	–	–	–	–	–	1.70^b^
	Salinger et al.^27^, ^a^^,^ d	153	0.85	–	–	–	–	–	–	–	–
4‐g loading + 2 g/hours continuous maintenance	Aali et al.^7^, ^e^	50	1.20 (0.30)	2.25 (0.75)	–	–	2.10 (0.85)	–	–	–	–
	Handwerker et al.^22^, ^d^	8	0.76 (0.05)	1.73 (0.14)	1.78 (0.13)	1.83 (0.13)	–	–	–	–	–
	Sibai et al.^5^, ^d^	7	–	–	1.86	1.87	1.94	2.00	2.10	–	1.84
	Taber et al.^32^, ^d^	9	0.90 (0.03)	–	–	–	2.38 (0.08)	–	–	–	1.99 (0.10)
	Tongsong et al.^33^, ^e^	25	–	–	–	2.27 (0.19)	2.33 (0.25)	–	–	–	–
5‐g loading + 1 g/hour continuous maintenance	Phuapradit et al.^26^, ^d^	44	0.95 (0.12)	1.97 (0.16)	1.93 (0.16)	1.85 (0.12)	1.97 (0.12)	–	2.22 (0.12)	2.42 (0.12)	–
6‐g loading + 2 g/hour continuous maintenance	Apostol et al.^13^, ^d^	16	–	–	–	–	–	–	–	–	2.05^b^
	Chissell et al.^16^, ^d^	8	0.80 (0.12)	1.90	1.75	1.70	1.75	1.80	1.80	–	–
	Guzin et al.^21^, ^e^	50	0.58 (0.21)	–	–	1.32 (0.26)	–	–	–	–	–
	Mason et al.^25^, ^d^	37	0.62 (0.01)	–	–	–	–	–	–	–	–
	Singh et al.^30^, ^e^	35	0.61 (0.17)	–	–	–	1.20	1.77	2.15	2.72	–
	Thurnau et al.^34^, ^d^	10	–	–	–	–	–	–	–	–	2.27^b^
4‐g loading + 2 g/hour intermittent bolus	Abbade et al.^12^, ^e^	14	0.74 (0.12)	1.23 (0.16)	1.11 (0.12)	1.03 (0.16)	1.07 (0.12)	–	–	–	–
4.5‐g loading + 1.8 g/hour continuous maintenance	Dayicioglu et al.^19^, ^d^	194	–	–	–	2.01 (0.33)	–	–	2.52 (0.43)	2.43 (0.34)	–
2‐g loading + 1.5 g/hour maintenance	Chesley and Tepper^3^, ^d^	8	0.90	–	1.27	1.44	1.56	–	–	–	–
2‐g loading dose only	Chesley and Tepper^3^ ^d^	3	0.74	–	0.95	0.90	–	–	–	–	–
4‐g loading dose only	Wright et al.^35^, ^d^	25	0.81 (0.10)	1.99 (0.18)^c^	–	–	–	–	–	–	–
120 mg/kg + 24 mg/kg × 5 hour	Lu et al.^23^, ^a^^,^ d	51	1.08 (0.21)	–	–	–	–	–	–	–	–
7.5–10 g/hour loading + 7.5–10 g over 4 hours	Chen et al.^14^, ^d^	30	0.92	–	2.16	–	–	–	–	–	2.16

SD, standard deviation.

a Model‐based study.

b Average concentration

c 16 minutes after initiating infusion.

d Data only for women with pre‐eclampsia.

e Data not disaggregated for women with pre‐eclampsia and eclampsia.

#### 4‐g loading dose and 2 g/hour continuous maintenance infusion

Five studies reported pharmacokinetic data based on the use of this regimen.^5^, ^8^, ^22^, ^32^, ^33^ Following administration of the loading dose, serum magnesium concentration rose rapidly to double the baseline values by ½ hour (1.73–2.25 mmol/l). Data on mean serum concentrations at 1, 2, 4, 8, and 12 hours from the start of infusion showed a gradual rise in serum magnesium to a plateau level, with mean levels slightly above 2.00 mmol/l being more consistent after 4 hours. The fluctuations in the serum magnesium levels were minimal and the described pattern was consistent with the steady state concentrations of 1.84 and 1.99 mmol/l, as reported by two studies.^5^, ^32^

Based on this regimen, one study estimated the volume of distribution of magnesium to be 16.40 l, plasma clearance to be 1.21 l/hour and elimination half‐life to be 20.2 hours.^32^ Three studies reported considerable variations in the ionised (free) magnesium fraction at baseline and during maintenance infusion.^8^, ^22^, ^32^ At baseline, the ionised fraction was between 50.0 and 64.9% of the total serum magnesium but these fractions appeared to decrease as the serum level approached steady‐state levels. Two of these studies demonstrated no correlation between ionised and total magnesium,^8^, ^32^ whereas the third study reported a strong correlation between ionised and total magnesium.^22^ This finding was supported by a report of a positive correlation of ionised and total magnesium in pre‐eclamptic women in another study.^36^ No other pharmacokinetic parameters were reported in the included studies.

#### 5‐g loading dose and 1 g/hour continuous maintenance infusion

Based on this regimen, one study reported that serum magnesium rose rapidly from baseline level of 0.95 mmol/l to 1.97 mmol/l by ½ hour, followed by a gradual decline by 1 hour before rising slowly again to steady between 2.20 and 2.42 mmol/l between 12 and 24 hours of maintenance infusion.^26^

#### 6‐g loading dose and 2 g/hour continuous maintenance infusion (Sibai regimen)

Six studies provided sparse pharmacokinetic data based on this regimen.^13^, ^16^, ^21^, ^25^, ^30^, ^34^ The reported baseline serum magnesium values were between 0.58 and 0.80 mmol/l.^16^, ^21^, ^25^, ^30^ Following initiation of MgSO4, one study showed that this level doubled in ½ hour before declining slightly to plateau between 1.70 and 1.80 mmol/l between 1 hour and 12 hours of maintenance infusion.^16^ At no point during the treatment did the serum level reach 2.00 mmol/l (peak concentration of 1.96 mmol/l was attained at 0.90 hour). Another study recorded a similar pattern of rapid rise and fall in serum magnesium following the loading dose but the mean levels gradually increased to achieve levels above 2.00 mmol/l between 12 and 24 hours.^30^ Two other studies also reported ‘average’ values of 2.05 and 2.27 mmol/l during administration of this regimen.^13^, ^34^

Two studies reported cerebrospinal fluid (CSF) magnesium levels of 1.23–1.34 mmol/l.^13^, ^34^ One study reported no significant alteration in the baseline ionised fraction of 53.6% in the CSF despite considerable increase in serum magnesium during administration of MgSO_4_ for periods of up to 48 hours.^13^ Baseline ionised magnesium in the serum was reported to be 69.8% in another study.^25^

#### Other intravenous regimens

Table 2 also shows the available data for seven less popular intravenous regimens. With 4‐g loading dose followed by 2 g/hour intermittent IV bolus injections, one study showed a rapid rise of serum magnesium from baseline value to a peak concentration of 1.64 mmol/l by 15 minutes, after which it fell very rapidly to 1.23 mmol/l by 2 hours.^12^ The first maintenance bolus dose at 2 hours was accompanied by another peak (1.69 mmol/l) and rapid fall to 1.07 mmol/l at 4 hours. For the most of the 4‐hour follow up of post‐dose serum concentration, the mean levels of magnesium remained around 1.00 mmol/l.

A study that administered a 4.5‐g loading dose and 1.8 g/hour maintenance dose reported a gradual serum magnesium rise from 2.01 mmo/l at 2 hours to a peak of 2.52 mmol/l at 12 hours before it declined to 2.43 mmol/l at 24 hours.^19^Another study evaluating the pharmacokinetics of lower intravenous regimen (2 g loading plus 1.5 g/hour continuous infusion) to women with eclampsia showed a very slow rise in mean serum magnesium levels from 1.27 at 1 hour to 1.56 mmol/l at 4 hours.^3^ Data (not shown) showed that the level remained sustained at <2 mmol/l by 6 hours of maintenance. The same study reported serum concentration‐time data for a 2‐g loading dose only for three eclamptic women.^3^ The study showed a transitory and trivial effect on the baseline magnesium—peaking within 10 minutes of injection and falling rapidly to 0.90 mmol by 2 hours.

One study estimated the initial volume of distribution based on a single intravenous injection of 4 g of MgSO_4_ to women with pre‐eclampsia to be 13.65 l.^25^Another study used an experimental weight‐based regimen (120 mg/kg loading dose and 24 mg/kg maintenance dose over 5 hours) and estimated the volume of distribution to be 49 l (central and peripheral) and total clearance to be 5 l/hour.^23^ Simulations based on the model developed by the same study showed that maintenance infusion rate of 1 g/hour produced concentrations well below 2.00 mmol/l and rarely produced concentrations >2.00 mmol/l during the first 10 hours of drug administration, whereas 2 g/hour infusion rate had higher probability of achieving concentrations within 2.00 and 4.00 mmol/l and lower probability of excess values.

Based on a regimen that included a 7.5–10 g loading dose, fast infusion over 1 hour and maintenance infusion of 7.5–10 g over 4 hours in 30 women with pre‐eclampsia, one study showed a rapid rise in magnesium level to over twice the baseline levels, reaching a steady state of 2.16 mmol/l.^14^

### Intramuscular MgSO4 regimens

#### 4‐g IV and 10‐g IM loading dose, and 5‐g IM maintenance dose every 4 hours (Pritchard regimen)

Six studies provided serum magnesium concentration‐time data based on this regimen.^5^, ^14^, ^18^, ^27^, ^28^, ^29^ Reported baseline magnesium levels were <1.00 mmol/l (Table 3). Following the loading dose, serum magnesium level rose sharply from the baseline to at least two‐fold by ½ hour (1.90–2.79 mmol/l). After the initial rise, Chissell et al. reported a slight decline in serum magnesium at 1 hour but relatively steady levels between 1.60 and 1.75 mmol/l until 12 hours of the maintenance injection.^16^ In the same study, serum level peaked at 2.07 mmol/l at 1½ hours following the initiation of treatment.

**Table 3:** Serum magnesium concentration‐time profile for intramuscular regimens

Regimen	Study	*n*	Mean serum magnesium in mmol/l (SD)								
			Baseline	½ hour	1 hour	2 hours	4 hours	8 hours	12 hours	24 hours	Steady state
4‐g (IV) + 10‐g IM loading + 5‐g 4 hourly maintenance	Chissell et al.^16^, ^a^	9	0.74	1.90	1.80	1.75	1.75	1.75	1.60	–	–
	Sibai et al.^5^, ^a^	8	–	2.79	2.79	2.63	2.10	1.97	1.93	–	2.71
	Singh et al.^30^, ^c^	35	0.64	–	–	–	1.36	1.85	2.14	2.59	–
	Ekele and Badung^20^, ^b^	19	0.72 (0.10)	–	–	–	1.90 (0.23)	2.21 (0.11)	2.18 (0.10)	–	–
	Shreya et al.^29^, ^b^	18	0.81 (0.10)	2.45 (0.43)	–	–	1.88 (0.51)	–	–	–	–
	Shreya et al.^29^, ^a^	22	0.78 (0.11)	2.60 (0.53)	–	–	1.97 (0.47)	–	–	–	–
3‐g (IV) loading + 10‐g (IM) maintenance	Chesley and Tepper^3^, ^a^	3	–	–	2.30	2.30	1.90	–	–	–	–
	Chesley^15^, ^a^	10	2.10	–	2.25	2.25	1.90	–	–	–	–
10‐g loading + 5 g (4 hourly) maintenance	Chesley and Tepper^3^, ^a^	Unspecified	–	–	1.36	1.56	1.48	–	–	–	–
	Sibai et al.^5^, ^a^	10	–	–	–	–	–	–	–	–	1.83
10‐g loading dose only	Chesley and Tepper^3^, ^a^	20	0.82	–	1.52	1.81	1.44	–	–	–	–
12‐g (2 g IV, 10 g IM) loading dose only	Seydoux et al.^28^, ^a^	5	0.80	–	–	–	–	–	–	–	1.50
4‐g IV, 4‐g IM loading dose only	Shreya et al.^29^, ^b^	9	0.73 (0.09)	1.94 (0.51)	–	–	1.49 (0.23)	–	–	–	–
	Shreya et al.^29^, ^a^	31	0.78 (0.15)	1.83 (0.35)	–	–	1.46 (0.27)	–	–	–	–

SD, standard deviation.

a Data for women only with pre‐eclampsia.

b Data for women only with eclampsia.

c Data not disaggregated for women with pre‐eclampsia and eclampsia.

Sibai et al.^5^ showed a rapid rise in the first 4 hours with values all above 2.00 mmol/l and a slight decline by 8 and 12 hours. Singh et al. reported a similar pattern with a gradual increase demonstrated between 4 and 24 hours following initiation of treatment.^30^ Ekele and Badung also showed a more than two‐fold rise in serum magnesium compared with baseline and mean levels slightly above 2.00 mmol/l at 8 and 12 hours of maintenance injections.^20^ Shreya et al. 29 also reported a similar increase in serum magnesium level in the first ½ hour and values slightly lower than 2.00 mmol/l at 4 hours. Overall, the serum‐concentration data fluctuated much more with this regimen than with continuous intravenous regimens described above, and serum level versus time data were less consistent across studies. However, for every time point reported, there were mean values reaching ≥2.00 mmol/l, but none reached 3.00 mmol/l.

Only one study reported on magnesium sulphate toxicity using this regimen. The study reported respiratory depression and death in a woman with a serum magnesium level of 9.90 mmol/l.^31^

#### 10‐g IM loading dose and 5‐g IM maintenance dose every 4 hours

Two studies reported serum‐concentration data based on this regimen.^3^, ^5^ In one of the studies, the mean levels of serum magnesium at 1, 2, and 4 hours were observed to be 1.36, 1.56, and 1.48 mmol/l, respectively.^3^ The other study only reported a steady state level of 1.83 mmol/l.^5^

#### 3‐g IV and 10‐g IM (13 g) loading dose only

With this regimen, one study reported a baseline serum magnesium of 2.10 mmol/l.^15^ The mean magnesium levels rose to 2.25 and 2.30 mmol/l at 1 and 2 hours following treatment, respectively, and gradually declined at 4 hours to 1.90 mmol/l.

#### Other intramuscular regimens

One study reported serum magnesium levels following the administration of a single 10‐g IM loading dose.^3^ Mean serum magnesium concentration rose slowly to attain its average peak of 1.81 mmol/l between 1½ and 2 hours before it declined to 1.44 and 1.34 mmol/l at 4 and 6 hours, respectively. Another study reported a steady‐state level of 1.50 mmol/l following a single 12‐g loading dose regimen.^28^ Using a single dose of 4 g MgSO_4_ intravenously and 4 g intramuscularly, another study reported that the mean serum level increased notably in the first ½ hour and then gradually decreased to levels similar to those achieved by the 10‐g IM loading dose‐only regimen.^29^

## Discussion

### Main findings

This review shows that the bioavailability for all intravenous regimens is complete and rapid as expected, and suggests a substantial bioavailability for the intramuscular regimens. Baseline serum magnesium concentrations were consistently <1 mmol/l. An intravenous loading dose of 4–6 g was associated with a rapid doubling of this baseline concentration within ½ hour of starting the injection. A maintenance infusion of 1 g/hour following a 4‐g loading dose (Zuspan regimen) consistently produced mean concentrations between 1 and 2 mmol/l throughout the period of administration. Maintenance infusion of 2 g/hour following either a 4‐ or a 6‐g loading dose had a higher likelihood of producing mean concentrations between 2 and 3 mmol/l with fewer fluctuations during the period of administration. Intermittent bolus injections of 2 g produced a spike in serum concentrations that fell very rapidly to almost basal levels within 2 hours of injection. The Pritchard regimen inconsistently produced serum concentrations between 2 and 3 mmol/l but the repeated intramuscular injections resulted in more fluctuations compared with continuous intravenous maintenance regimens. The volume of distribution of magnesium varied significantly but plasma clearance was fairly similar across populations.

### Strengths and limitations

To our knowledge, this is the first systematic review of clinical pharmacokinetic properties of MgSO_4_ when used for prevention and treatment of eclampsia. We minimised potential bias in the review process by searching major databases without language or date restrictions to capture all relevant studies as far back as the 1950s.

The main limitation of this review was the inclusion of pharmacokinetic parameters from different study designs with varying primary objectives and study characteristics. However, we minimised potential bias by avoiding meta‐analysis and performing descriptive syntheses of available pharmacokinetic data, thus taking advantage of the available knowledge on the subject without compromising methodological rigour of a systematic review. Another limitation is the inclusion of studies that combined data of women with pre‐eclampsia and eclampsia, which precluded a clear description of pharmacokinetics according to disease severity. Nevertheless, the proportion of women with eclampsia in the included studies is unlikely to impact significantly on the patterns described.

### Interpretations

Although there is no consensus on the magnesium concentration required to prevent or treat eclamptic seizures, serum concentrations between 2 and 3.5 mmol/l (4–7 mEq/l) are generally held to be therapeutic and have directly and indirectly driven clinical practice for decades.^6^ However, there is no evidence from our review that concentrations within this range are consistently achieved even by the two most popular and clinically efficacious regimens (Zuspan and Pritchard).^1^, ^2^ This suggests that the minimum effective serum magnesium concentration is likely to be lower and the therapeutic window wider than generally accepted levels.

Of particular interest is the maintenance dose of the Zuspan regimen, which was associated with a steady‐state level that was well below 2 mmol/l. Similar observations have led some researchers to advocate higher intravenous loading and maintenance doses to match serum levels achieved by the Pritchard regimen in order to increase therapeutic efficacy.^27^ It is important to note, however, that a substantial proportion of women receiving the Pritchard regimen are also below the ‘therapeutic range’ at varying time points during the course of treatment. Interestingly, the clinical efficacy in terms of seizure prophylaxis for both regimens is generally considered comparable.^1^, ^2^ As higher doses risk excessive serum magnesium levels and toxicity, the noted differences in the associated serum concentrations do not justify a review of the Zuspan dosing regimen.

Our findings highlight the disconnection between the pharmacokinetics and choice of alternative MgSO_4_ regimens for treating women with pre‐eclampsia and eclampsia. For instance, the use of a 2‐g loading dose only or a 2 g/hour bolus IV injection only produces transient and trivial effects on the serum magnesium concentrations and is unlikely to be clinically effective. On the other hand, the review provides support for the clinical non‐inferiority shown by some regimens. For example, the fact that the 10‐g IM loading dose‐only regimen produced serum levels similar to the Zuspan regimen for up to 6 hours may explain the reported comparative efficacy of Pritchard regimens in settings where prompt delivery of women with pre‐eclampsia and eclampsia is possible.

## Conclusions

It would be useful to consider what is known about the basic pharmacokinetic profile of MgSO_4_ when selecting dosage regimens of unproven efficacy for clinical use. As evident from the serum levels attained during treatment with the Zuspan regimen, it appears that MgSO_4_ can be protective even with serum concentrations of <2 mmol/l. Therefore, titrating MgSO_4_ injections to achieve a pre‐set therapeutic range of 2–3.5 mmol may risk toxic levels without necessarily improving clinical protection against seizures. Regardless of the slight differences in the pharmacokinetic profiles of the two currently recommended regimens, the comparability of their clinical efficacy is reassuring and does not justify a further increase in the total dose of MgSO_4_ for prophylaxis and treatment of eclampsia. The single or intermittent use of intravenous bolus injection of 2 g MgSO_4_ does not produce a sustained increase in serum magnesium levels to provide a clinically meaningful protection against seizures and is best avoided.

Most of the studies included in this review had small numbers of participants and the majority did not report on the full pharmacokinetic properties of MgSO_4_. In future research, efforts should be made to investigate the complete profile for any particular regimen with separate data for pre‐eclampsia and eclampsia. The demonstrated serum magnesium levels achieved by clinically efficacious regimens can be targeted in standard pharmacokinetic‐pharmacodynamic (PK/PD) modelling and simulation studies to determine the minimum effective dosage of MgSO4 for prophylaxis and treatment of eclampsia.

### Disclosure of interests

None declared. Completed disclosure of interests form available to view online as supporting information.

### Contribution to authorship

OTO and AMG conceived the study. OTO prepared the study protocol with input from AMG and BOO. BOO and OTO performed initial screening of search outputs, identified eligible studies and extracted data. QL updated the search and along with BOO, screened the updated search outputs for eligible studies. BOO and OTO drafted the manuscript. BOO, OTO, QL, PL, GC, ZQ, LD, JPS, and AMG interpreted the data and revised the manuscripts for intellectual contents. All authors approved the manuscript for publication.

### Details of ethics approval

No ethical approval was required for this study.

### Funding

The study was supported by a grant from Merck, through its Merck‐for‐Mothers Programme, to UNDP/UNFPA/UNICEF/WHO/World Bank Special Programme of Research, Development and Research Training in Human Reproduction (HRP), Department of Reproductive Health and Research, World Health Organization. The funder was not involved in the design, data collection, analysis, interpretation or writing of this report, nor the decision to submit the article for publication.

## Supporting information


**Figure S1.** Risk of bias assessment of the included studies.

**Figure S2.** Risk of bias assessment for intravenous regimens.

**Figure S3.** Risk of bias assessment for intramuscular regimens.

**Table S1.** Checklist for risk of bias assessment and explanations.

**Table S2.** Characteristics of included studies.

**Table S3.** Characteristics of excluded studies and reasons for exclusion.

**Table S4.** Risk of bias assessment of included studies.

**Box S1.** References to excluded studies.
