# Supplementary material for: Clinical pharmacokinetic properties of magnesium sulphate in women with pre‐eclampsia and eclampsia
Source: BJOG. 2015 Nov 24;123(3):356–66. doi: 10.1111/1471-0528.13753 (PMC4737322; doi:10.1111/1471-0528.13753)
Supplement: Supplementary file 1 — Figure S1. Risk of bias assessment of the included studies. [file BJO-123-356-s001.docx]

| **Figure S1. Risk of bias assessment of the included studies** | | | | | | | | |
| --- | --- | --- | --- | --- | --- | --- | --- | --- |
|  | Number of participants and MgSO4 sampling | Patients representativeness | Covariates | Study objective alignment with the systematic review objective | Laboratory method detail | Technology of test unchanged since the study | Baseline and duration of post-dose estimation | Withdrawals explained |
| Aali et al. ^8^ | + | + | ? | + | + | + | + | ? |
| Abbade et al. ^12^ | + | ? | ? | + | ? | ? | - | + |
| Apostol et al. ^13^ | + | ? | ? | + | - | ? | ? | + |
| Chen et al. ^14^ | + | + | ? | + | ? | ? | ? | ? |
| Chesley and Tepper ^3^ | + | ? | - | + | ? | ? | ? | ? |
| Chesley ^15^ | - | ? | - | - | ? | ? | ? | + |
| Chissell et al. ^16^ | + | + | ? | + | ? | ? | ? | + |
| Chuan et al. ^17^ | + | + | ? | + | + | ? | ? | + |
| Cruikshank et al. ^18^ | + | ? | - | - | + | + | + | ? |
| Dayicioglu et al. ^19^ | + | + | ? | + | ? | ? | ? | + |
| Ekele and Badung ^20^ | + | ? | ? | + | ? | ? | ? | + |
| Guzin et al. ^21^ | + | + | - | - | - | ? | ? | ? |
| Handwerker et al. ^22^ | + | + | ? | + | + | ? | ? | ? |
| Lu et al. ^23^ | + | + | - | - | - | ? | ? | ? |
| Manorot et al. ^24^ | + | ? | ? | + | ? | ? | ? | ? |
| Mason et al. ^25^ | + | ? | - | - | + | ? | ? | ? |
| Phuapradit et al.^26^ | + | ? | ? | + | + | + | ? | ? |
| Salinger et al. ^27^ | + | ? | ? | + | ? | ? | ? | + |
| Seydoux et al. ^28^ | + | + | - | + | ? | + | ? | + |
| Shreya et al. ^29^ | + | + | ? | + | - | ? | ? | + |
| Sibai et al. ^5^ | + | + | + | + | + | + | ? | + |
| Singh et al. ^30^ | + | + | ? | + | ? | + | + | ? |
| Suvarna et al. ^31^ | + | - | ? | - | - | ? | - | + |
| Taber et al. ^32^ | + | ? | ? | + | + | ? | ? | + |
| Tongsong et al. ^33^ | + | ? | + | + | ? | + | - | + |
| Thurnau et al. ^34^ | + | + | ? | - | ? | ? | - | + |
| Wright et al. ^35^ | + | + | ? | + | ? | ? | - | + |
| Yoshida et al. ^36^ | + | + | ? | + | ? | ? | ? | ? |
