# Supplementary material for: Clinical pharmacokinetic properties of magnesium sulphate in women with pre‐eclampsia and eclampsia
Source: BJOG. 2015 Nov 24;123(3):356–66. doi: 10.1111/1471-0528.13753 (PMC4737322; doi:10.1111/1471-0528.13753)
Supplement: Supplementary file 2 — Figure S2. Risk of bias assessment for intravenous regimens. [file BJO-123-356-s002.pdf]

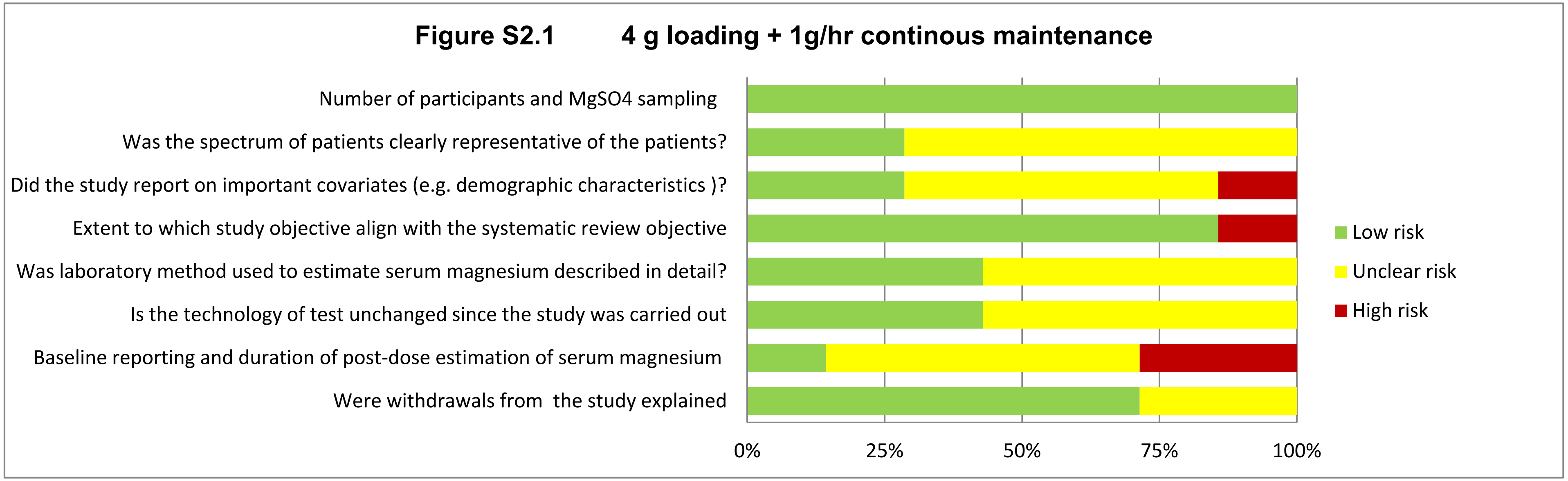
Figure S2.1 4 g loading + 1g/hr continous maintenance
Number of participants and MgSO4 sampling
Was the spectrum of patients clearly representative of the patients?
Did the study report on important covariates (e.g. demographic characteristics)?
Extent to which study objective align with the systematic review objective
Was laboratory method used to estimate serum magnesium described in detail?
Is the technology of test unchanged since the study was carried out
Baseline reporting and duration of post-dose estimation of serum magnesium
Were withdrawals from the study explained
0%
25%
50%
75%
100%
Low risk
Unclear risk
High risk

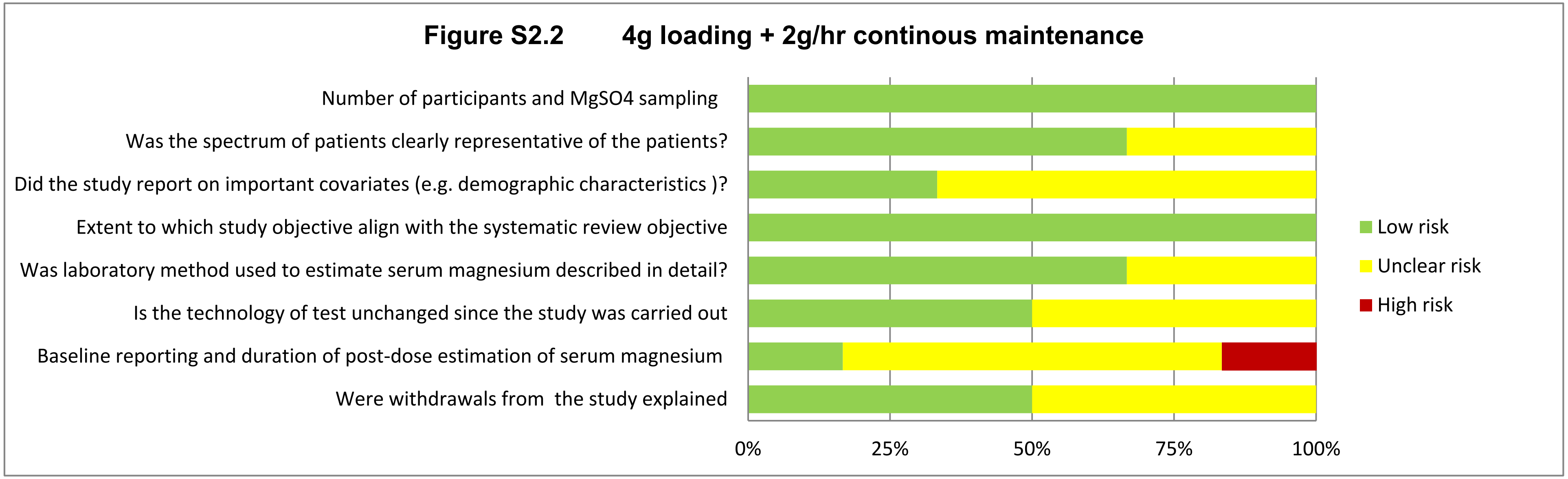
Figure S2.2 4g loading + 2g/hr continous maintenance
Number of participants and MgSO4 sampling
Was the spectrum of patients clearly representative of the patients?
Did the study report on important covariates (e.g. demographic characteristics)?
Extent to which study objective align with the systematic review objective
Was laboratory method used to estimate serum magnesium described in detail?
Is the technology of test unchanged since the study was carried out
Baseline reporting and duration of post-dose estimation of serum magnesium
Were withdrawals from the study explained
0%
25%
50%
75%
100%
Low risk
Unclear risk
High risk

Figure S2.3 5g loading + 1g/hr continous maintenance

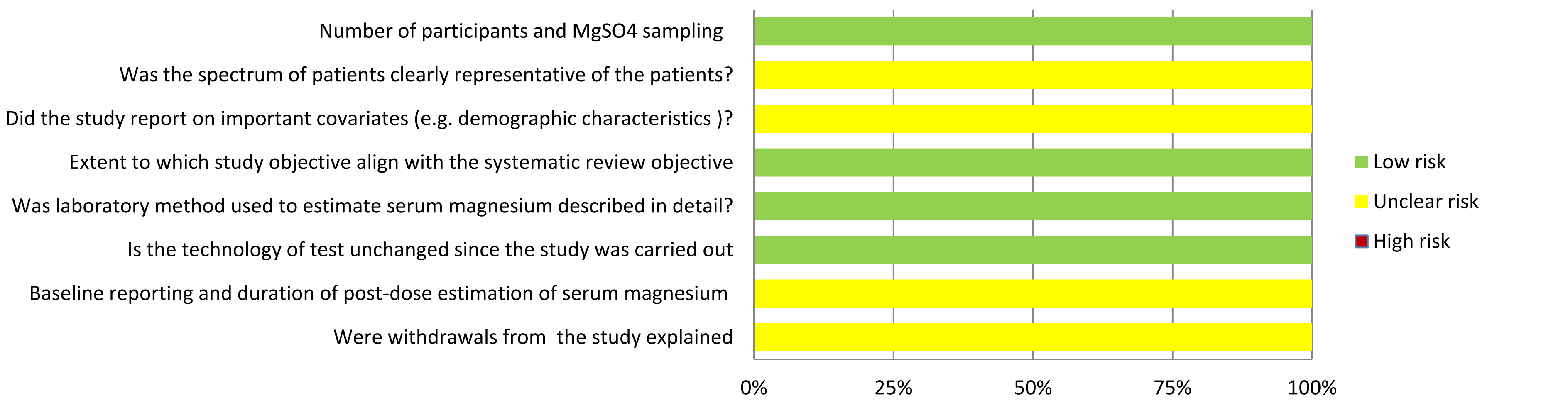

Figure S2.4 6g loading + 2g/hr continous maintenance

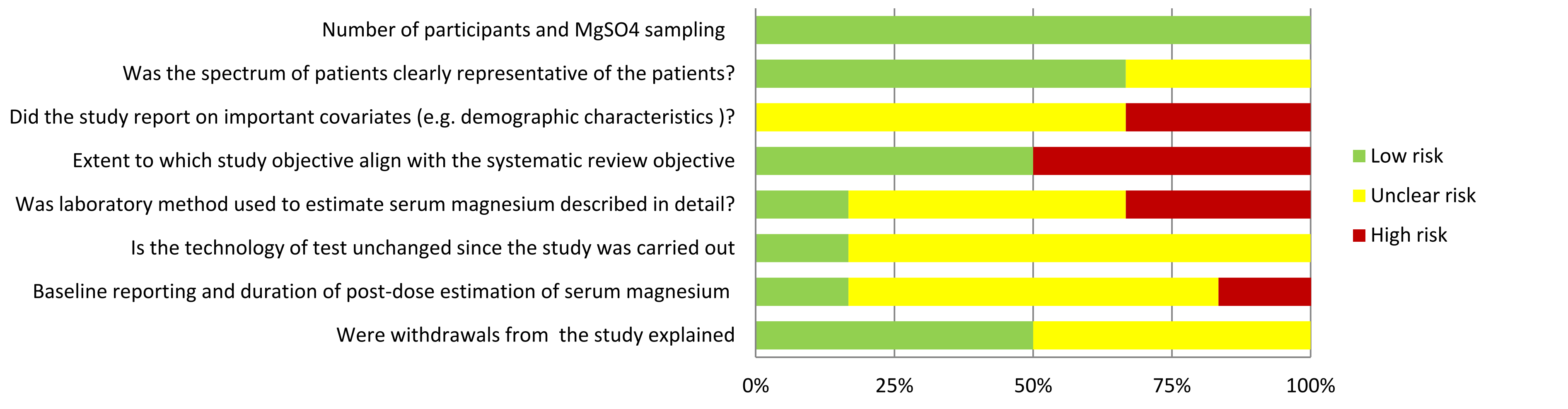

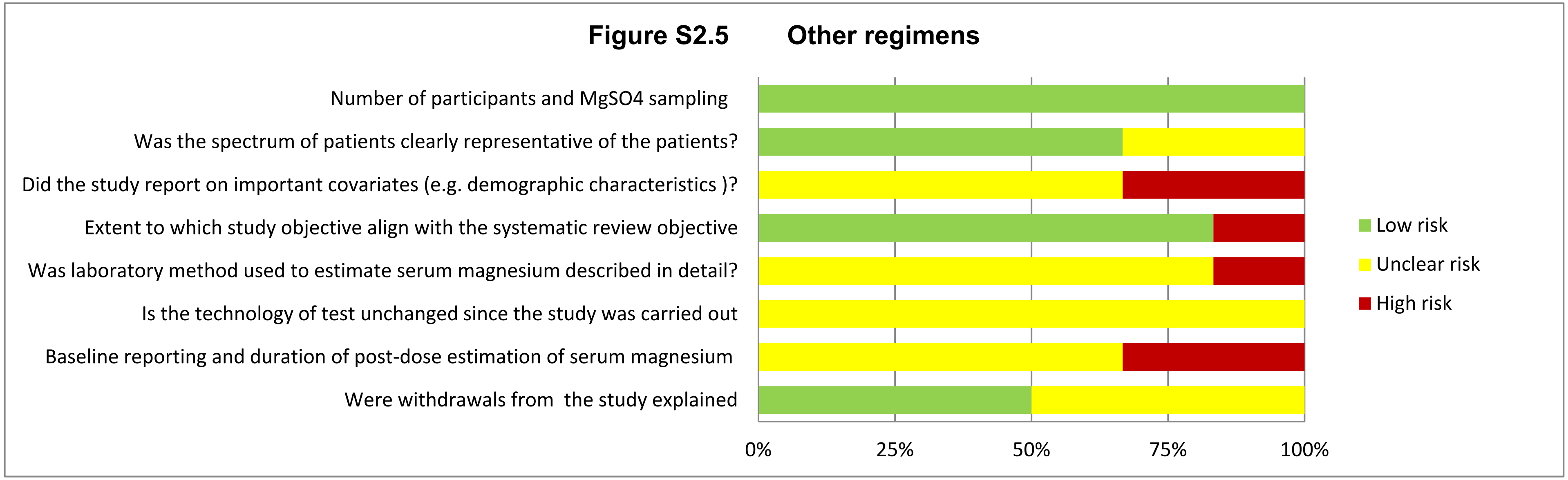

Figure S2. Risk of bias assessment for intravenous regimens
