# Supplementary material for: Clinical pharmacokinetic properties of magnesium sulphate in women with pre‐eclampsia and eclampsia
Source: BJOG. 2015 Nov 24;123(3):356–66. doi: 10.1111/1471-0528.13753 (PMC4737322; doi:10.1111/1471-0528.13753)
Supplement: Supplementary file 3 — Figure S3. Risk of bias assessment for intramuscular regimens. [file BJO-123-356-s003.pdf]

Figure S3.1 4g (IV) + 10 g IM loading + 5g 4 hrly maintenance

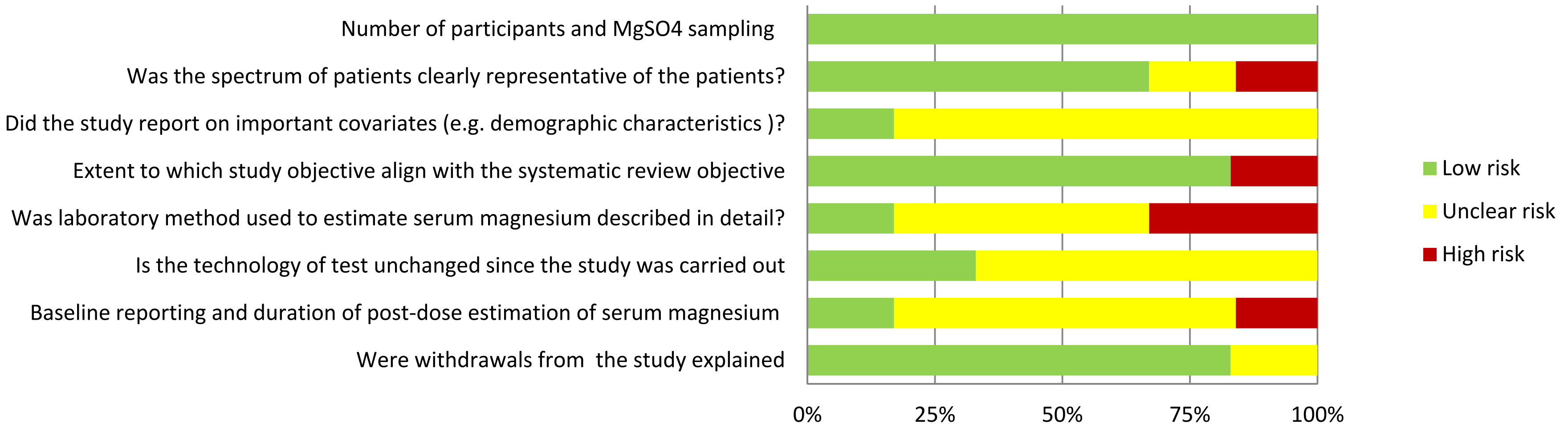

Figure S3.2 3g (IV) loading + 10g (IM) maintenance

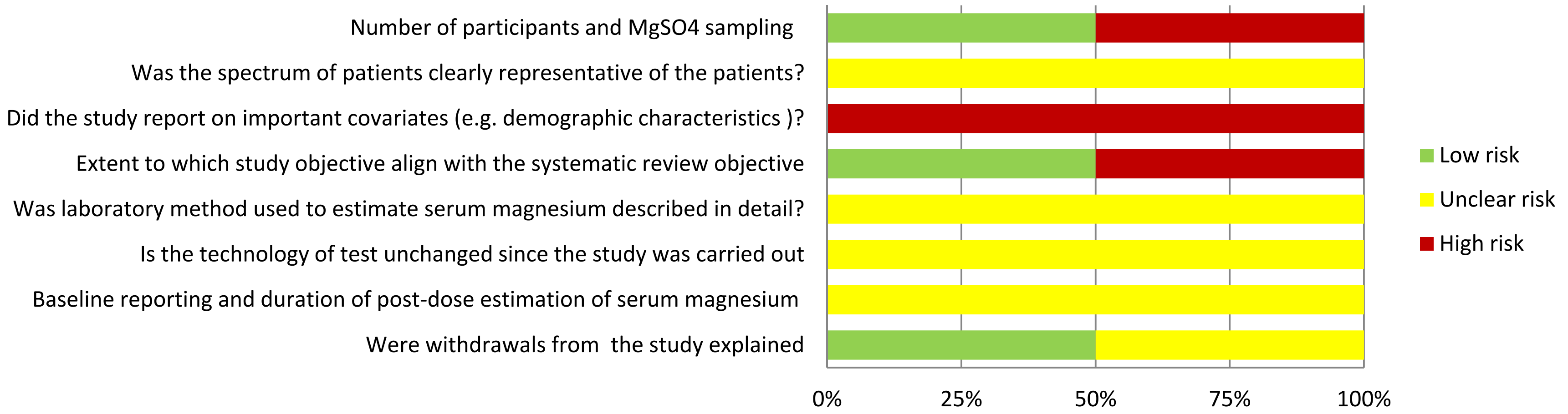

Figure S3.3 10 g loading + 5 g (4hrly) maintenance

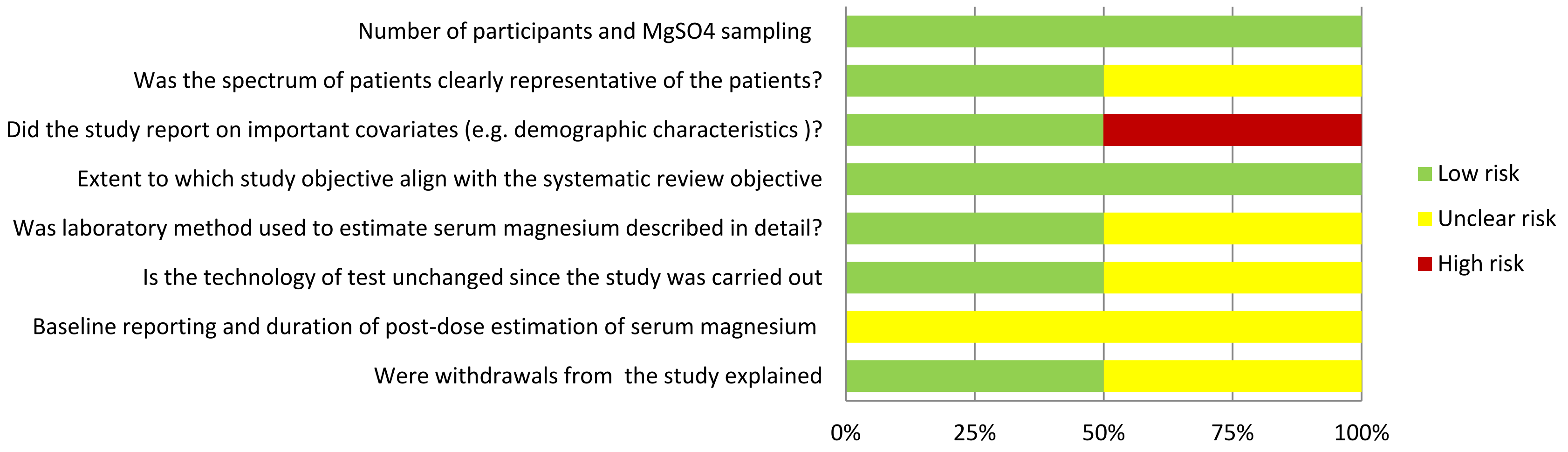

Figure S3.4 10g loading dose only

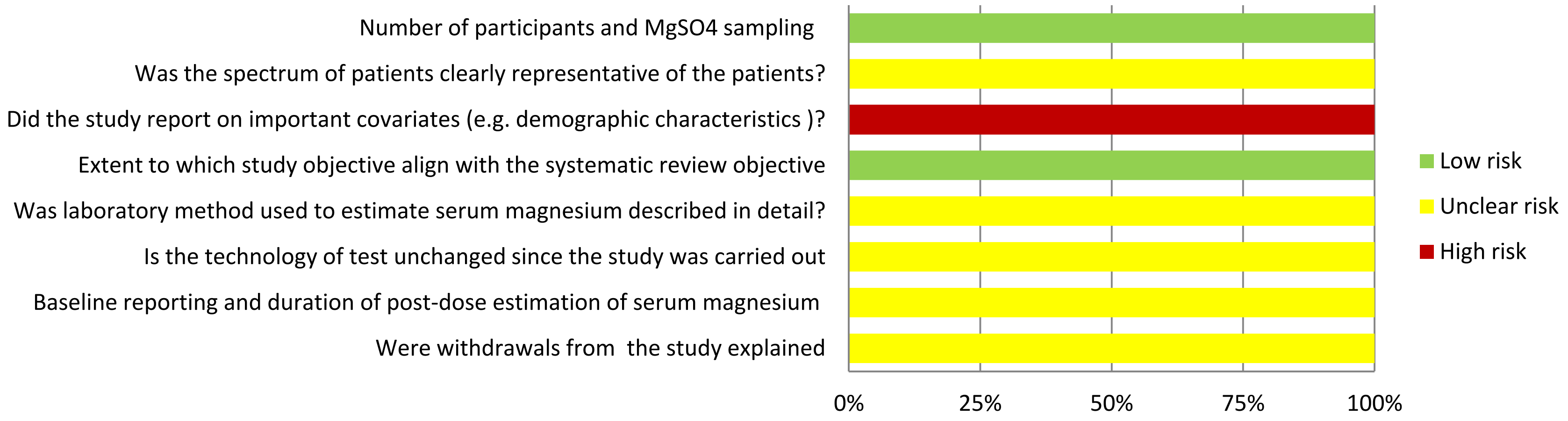

Figure S3.5 12g (2g IV, 10g IM) loading dose only

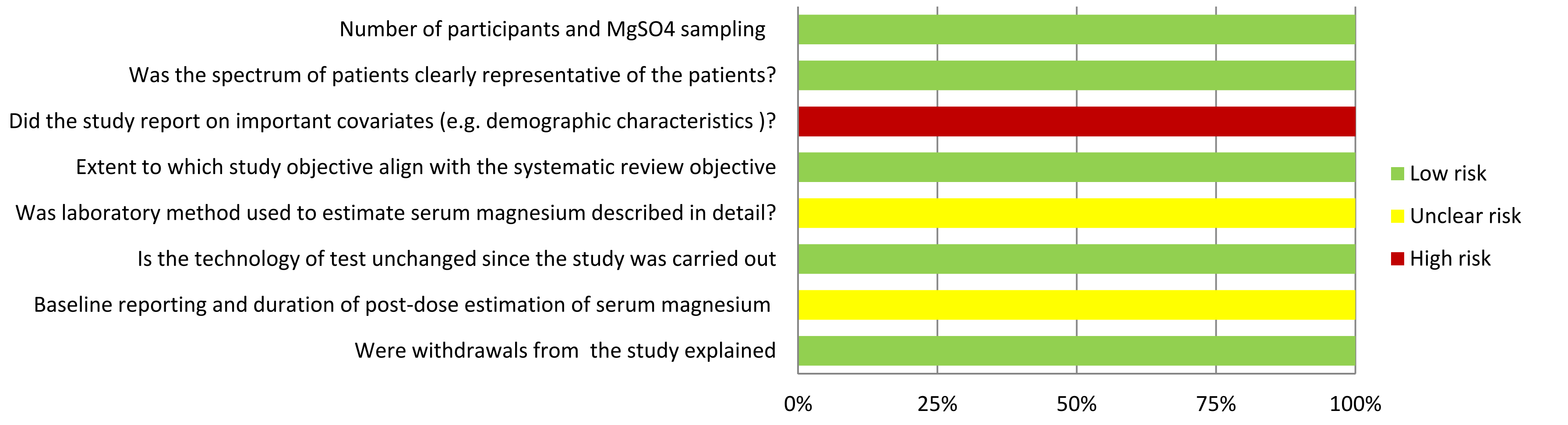

Figure S3.6 4g IV and 4g IM loading dose only

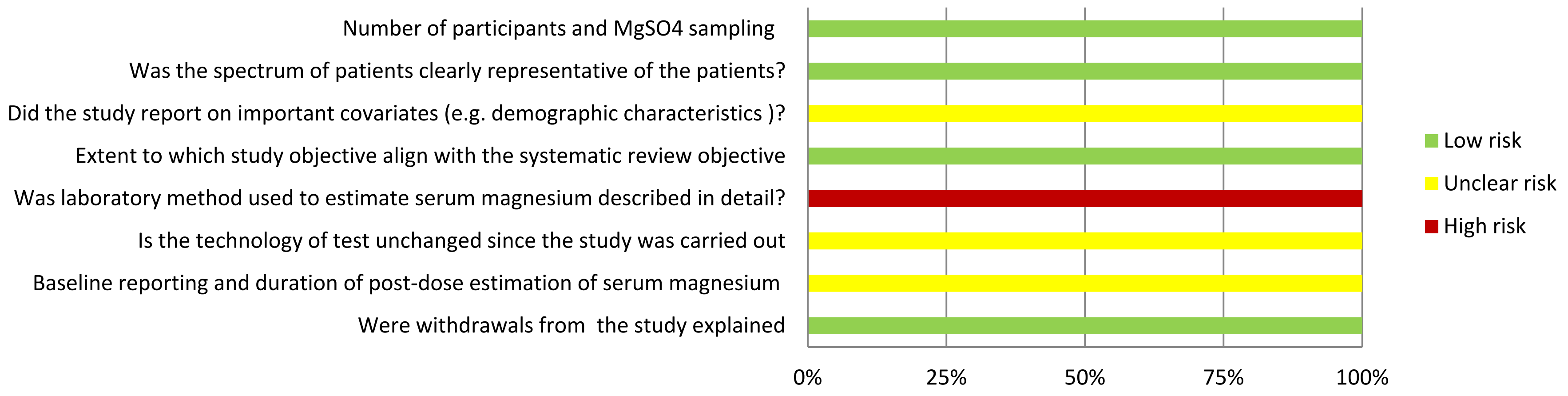

Figure S3. Risk of bias assessment for intramuscular regimens
