# Supplementary material for: Clinical pharmacokinetic properties of magnesium sulphate in women with pre‐eclampsia and eclampsia
Source: BJOG. 2015 Nov 24;123(3):356–66. doi: 10.1111/1471-0528.13753 (PMC4737322; doi:10.1111/1471-0528.13753)
Supplement: Supplementary file 4 — Table S1. Checklist for risk of bias assessment and explanations. [file BJO-123-356-s004.pdf]

Table S1. Checklist for risk of bias assessment and explanations

Study	Domain of assessment	Criteria	Risk of bias	Explanation
	**Number of participants and MgSO4 sampling**	≥10 or dense sampling in ≥5	Low	≥10 women treated with $MgSO_4$ for preeclampsia and/or eclampsia with serum magnesium concentration measured against time **OR** ≥5 women with dense sampling per subject.
		<10 or sparse sampling in ≥5-9	High	<10 women treated with $MgSO_4$ for preeclampsia and/or eclampsia with serum magnesium concentration measured against time **OR** <5 women with dense sampling per subject.
		Unclear	Unclear	Number of participants with serum magnesium estimation was not reported.
	**Was the spectrum of patients clearly representative of the patients who will receive the test in practice?**	Yes	Low	The study used criteria that are accepted to define preeclampsia or eclampsia or made reference to the criteria.
		No	High	The study did not use criteria that are accepted to define preeclampsia or eclampsia or make reference to the criteria.
		Unsure	Unclear	Study reported use of $MgSO_4$ for women with “toxaemia”, “preeclampsia” and / or “eclampsia” without any reference to clear criteria for selecting study participants.
	**Did the study report on important covariates (e.g. demographic characteristics - maternal age, gestation age, race,**	Yes	Low	Details of relevant covariates were provided in the report for all women or as a range.
		No	High	No report of relevant covariates provided in the report.
		Unclear	Unclear	Covariate sparsely reported for women or described as arithmetic mean or

**body weight or body mass index)?**			percentages.
**Extent to which study objective align with the systematic review objective**	Yes	Low	The primary objective of the study was to assess serum level magnesium concentrations or pharmacokinetic properties of $MgSO_4$ in women with preeclampsia and/ or eclampsia. It covers Absorption, Distribution, Metabolism, Excretion (ADME).
	No	High	The primary objective of the study was not to assess serum magnesium level or pharmacokinetic properties of $MgSO_4$ in preeclampsia and/or eclampsia but serum Mg concentration was assessed as part of the study components.
	Uncertain	Unclear	The study objective was not clearly reported and does not fall into any of the above categories.
**Was laboratory method used to estimate serum magnesium described in detail?**	Yes	Low	Sample collection explained including storage conditions of samples prior to estimation of serum magnesium. The equipment, model, manufacturer and peculiarities of laboratory method described.
	No	High	No description of method of serum magnesium estimation.
	Unclear	Unclear	Partial description of the storage conditions of samples prior to estimation of serum magnesium, the equipment, model, manufacturer and or the peculiarities of laboratory method.
**Is the technology of test unchanged since the study was carried out**	No	Low	Method and equipment used to estimate serum magnesium concentration have not changed since study was conducted.
	Yes	High	Method and equipment used to estimate serum magnesium have changed since study was conducted.
	Unsure	Unclear	Unable to ascertain if technique and equipment used to estimate serum magnesium have not changed since study was conducted
**Baseline reporting and**	Baseline reported and	Low	Baseline and post-dose serial serum magnesium concentration reported against time for up to 24 hours (i.e. sampling covers absorption phase,

**duration of post-dose estimation of serum magnesium**	post-dose estimation ≥24 hours		maximum concentration region and elimination phase).
	No baseline reported or post-dose estimation < 24 hours	High	Baseline not reported or post-dose serial serum magnesium concentration reported against time for less than 24 hours (i.e. sampling does not cover absorption phase, maximum concentration region and elimination phase).
	Unreported or unclear reporting	Unclear	Baseline serum magnesium was not reported and post-dose serial measurement was not performed up to 24 hours OR baseline serum magnesium was reported but post-dose serial measurement was not performed up to 24 hours.
Were withdrawals from the study explained	Yes/ No withdrawals	Low	Less than 20% withdrawal or explanation suggests that withdrawals would not bias the study findings
	No	High	More than 20% withdrawal or explanation suggests that withdrawals would bias the study findings.
	Unclear	Unclear	No explanation provided for withdrawals.
