# Supplementary material for: Clinical pharmacokinetic properties of magnesium sulphate in women with pre‐eclampsia and eclampsia
Source: BJOG. 2015 Nov 24;123(3):356–66. doi: 10.1111/1471-0528.13753 (PMC4737322; doi:10.1111/1471-0528.13753)
Supplement: Supplementary file 5 — Table S2. Characteristics of included studies. [file BJO-123-356-s005.docx]

**Supplementary table S2: Characteristics of included studies**

| **Aali et al.^8^** | **Characteristics** |
| --- | --- |
| **Study design** | Cross-sectional study. |
| **Study objective** | To determine the correlation between ionized and total magnesium under baseline and therapeutic conditions |
| **Participants** | 50 women: 46 women with severe pre-eclampsia and four women with eclampsia. Severe pre-eclampsia was defined as blood pressure ≥ 160/110 mmHg and proteinuria ≥ 2+. Eclampsia was defined as convulsion in a woman with pre-eclampsia not attributable to other causes. Most participants were young with mean age of 27.5 years and mean weight of 80.8 kg.  Exclusion criteria: not stated  Setting: Maternity Centre of Afzalipour Hospital, Kerman City, South-East Iran. |
| **Dosage regimen** | 4 g intravenous loading dose and 2 g continuous intravenous maintenance dose of MgSO_4_ until 24 hours after delivery or 24 hours after the last seizure. |
| **Outcomes** | Serum ionized and total magnesium (mEq/L) |
| **Laboratory method of estimating serum magnesium concentration** | Serum total magnesium was estimated with an atomic absorption spectroscope (Shimadzu 620. Tokyo Japan) while ionized magnesium was analyzed by dye-binding colorimetry in which magnesium ion forms a red complex with Eriochrom black-T in alkaline solution (Shimadzu-2100). |
| **Notes** | Data for women with severe pre-eclampsia and eclampsia were not separated. |

| **Abbade et al.^12^** | **Characteristics** |
| --- | --- |
| **Study design** | Randomized controlled trial. |
| **Study objective** | To determine whether magnesium serum concentrations in patients with severe pre-eclampsia or eclampsia treated with two different regimens were different. |
| **Participants** | 29 women: 28 with pre-eclampsia and one woman with eclampsia. There were 23 white and six black participants with mean gestational age of 34.5 weeks, mean age of 28.6 years and mean weight of 74.8 kg. The definition of pre-eclampsia was not reported.  Exclusion criteria: Women with seizures other than eclampsia, laboratory evidence of reduced renal function and oliguria.  Setting: University Hospital of the Botucatu School of Medicine of Sao Paulo State University, Sao Paulo, Brazil. |
| **Dosage regimen** | All women had 4 g intravenous loading dose of MgSO_4_ over 15-20 minutes. Thereafter, a group had 1 g/hour continuous maintenance infusion (Zuspan regimen) while the other group had 2 g in bolus (intermittent) intravenous injection of MgSO_4_ every 2 hours. Blood samples were collected every 15 minutes over four hours after treatment. |
| **Outcomes** | Comparison of plasma magnesium serum concentrations in both regimen. |
| **Laboratory method of estimating serum magnesium concentration** | Serum magnesium was determined by Johnson and Johnson Vitros 950 dry-chemistry colorimetric analyzer. |
| **Notes** | The mean serum magnesium level was reported for all women irrespective of the indication for MgSO_4_. However, only one woman had eclampsia. |

| **Apostol et al.^13^** | **Characteristics** |
| --- | --- |
| **Study design** | Cross-sectional study. |
| **Study objective** | To correlate magnesium levels of serum with those of CSF in pre-eclamptic women receiving IV MgSO_4_ in therapeutic doses and determine whether magnesium crosses the blood-brain barrier after IV MgSO_4_. |
| **Participants** | 16 women with pre-eclampsia. Pre-eclampsia was diagnosed using the “classic criteria: hypertension, edema and proteinuria”. All subjects were African-American older than 20 and less than 35 years with a mean age of 25 ± 5 years. No information on participants’ weight was provided.  Exclusion criteria: History of neurologic disease, renal disease, hypertension, vascular disease and preterm labour.  Setting: Department of Obstetrics and Gynecology, Downstate Medical Center, Brooklyn, New York, USA. |
| **Dosage regimen** | Intravenous 6 g loading dose over 15-20 minutes followed by 2 g/ hour maintenance dose. |
| **Outcomes** | Distribution of ionized and total magnesium in serum and cerebral spinal fluid (CSF) in pre-eclamptic women and effects on calcium and ionized Ca: Mg ratios. |
| **Laboratory method of estimating serum magnesium concentration** | CSF was collected at the induction of spinal anaesthesia for all pregnant women and 3-ml sample of blood was collected at the same time. Total Mg was measured by “standard techniques” (Kodak DT 60; Ektachem Colorimetric Instruments, Rochester, NY). Blood for measurement of ionized magnesium was drawn anaerobically and its serum stored at -4 degree Celsius before measurement was done with Nova 8 analyzer; Nova Bio-chemical, Waltham, MA |
| **Notes** | None. |

| **Chen et al.^14^** | **Characteristics** |
| --- | --- |
| **Study design** | Open label study design. |
| **Study objective** | To develop population PK models for MgSO_4_ in women with pregnancy-induced hypertension and to investigate the associated blood-pressure lowering effect. |
| **Participants** | 30 women with pregnancy-induced hypertension defined as blood pressure 140-190 mmHg/100-120 mmHg, urinary protein ++ to ++++, oedema ++, with headache, vomiting or nausea, but with normal kidney function). Most participants were young, range 24-36 years old.  Exclusion criteria: Not stated  Setting: General Hospital of Nanjing Armed Forces, Nanjing, P.R. China. |
| **Dosage regimen** | Fast IV infusion 7.5-10 g in 1 hour followed by slow IV infusion 7.5-10 g in 4 hours and then doses adjusted based on the patient’s performance. Total of 15-20 g/24 hours. |
| **Outcomes** | Serum magnesium (mmol/L): Mean/SD at baseline, end of fast infusion, end of slow infusion, and 2, 4, 6 and 10 hours after the end of slow infusion.  Population 1 compartment PK model: K, t_1/2_ and Vc parameters estimates.  Effect on blood pressure: Mean/SD at baseline, end of fast infusion, end of slow infusion, and 2, 4, 6 and 10 hours after the end of slow infusion. |
| **Laboratory method of estimating serum magnesium concentration** | Magnesium levels were estimated using colorimetric end-points at selected time points (including baseline). Linear range (12-72 µg/ml). Precision CV% <2%. Recovery% 100.6 ± 1.1%. |
| **Notes** | None. |

| **Chesley and Tepper ^3^** | **Characteristics** |
| --- | --- |
| **Study design** | Cross-sectional study. |
| **Study objective** | To know what plasma magnesium levels would follow various doses of magnesium sulfate, given in various ways. |
| **Participants** | 52 women with either pre-eclampsia or eclampsia. Definition of pre-eclampsia not given but authors included cases of mild pre-eclampsia (about half of participants). Information on participants’ age, weight and gestational age at administration of MgSO_4_ was not provided.  Exclusion criteria: Not stated  Setting: Department of Obstetrics and Gynecology, State University of New York College of Medicine and Kings County Hospital, Brooklyn, New York, USA. |
| **Dosage regimen** | Different doses of MgSO_4_:   - Intravenous 2 g loading dose followed by 25 mg/min maintenance dose - Intravenous 2 g loading dose - Intravenous 3 g loading + 10g (IM) maintenance - Intramuscular 10 g loading + 5 g (4hrly) maintenance - Intramuscular 10 g loading dose |
| **Outcomes** | Serum levels of magnesium with in various doses |
| **Laboratory method of estimating serum magnesium concentration** | Blood samples taken at intervals had serum magnesium measured in duplicates for quality control. Serum magnesium was measured by the method of Simonsen, Westover and Wertman using the Beckman DU spectrophotometer |
| **Notes** | Results of the different doses were presented in Concentration-Time Curves from which the average mean serum level of magnesium ion was extracted. Also, the number of women who had 10 g intramuscular (IM) loading dose and a single 5 g IM maintenance dose was not stated. |

| **Chesley ^15^** | **Characteristics** |
| --- | --- |
| **Study design** | Comparative study. |
| **Study objective** | To indicate that the initial dose of 3 g intravascular and 10 g intramuscular is safe because of the volume in which it is diluted. |
| **Participants** | 3 women with pre-eclampsia. The definition of pre-eclampsia and the demographic characteristics of participants were not provided.  Exclusion criteria: Not stated  Setting: Department of Obstetrics and Gynaecology, State University of New York, Downstate Medical Centre and Kings County Hospital, Brooklyn, New York, USA. |
| **Dosage regimen** | 3 g intravenous and 10 g intramuscular (13 g) loading dose of MgSO_4_. |
| **Outcomes** | Measurement of the apparent volume of distribution of sucrose and magnesium. |
| **Laboratory method of estimating serum magnesium concentration** | Magnesium was measured by the method of Simonsen and co-workers in undiluted serum. |
| **Notes** | Results of apparent volume of distribution in three pre-eclamptic women marred by “equilibrium not established in two women while the third woman had lost 10 pounds (4.5 kg) in 5 days due to treatments with diuretics (acetazolamide and chlorthiazide)”. |

| **Chissell et al.^16^** | **Characteristics** |
| --- | --- |
| **Study design** | Cross-sectional study. |
| **Study objective** | To compare the clinical outcome as well as the magnesium levels obtained in the treatment of severe pre-eclampsia with IM regimen of Pritchard and IV regimen with maintenance doses of 2 g/h. |
| **Participants** | 17 women with severe pre-eclampsia and imminent eclampsia defined as proteinuria of at least 1+ on dipstick method (Ames) and diastolic blood pressure of ≥ 110mmHg. Imminent eclampsia was defined persistent headaches, visual disturbance, epigastric pain, increased patellar reflexes and clonus, in addition to hypertension and proteinuria. Participants’ average age, gestational age and weight was 22.4 years, 35.3 weeks and 73.7 kg respectively.  Exclusion criteria: Not stated  Setting: Department of Obstetrics and Gynaecology, King Edward VII (KEH) Hospital, Durban, South Africa. |
| **Dosage regimen** | Pritchard regimen (14 g loading dose and 5 g IM maintenance dose every 4 hours) was compared with 6 g intravenous loading dose and 2 g intravenous maintenance continuous infusion |
| **Outcomes** | Clinical outcome and magnesium levels with the different regimens. |
| **Laboratory method of estimating serum magnesium concentration** | Blood was taken from a central venous pressure line and samples analyzed the following day using dye-binding procedure with calmagite (Beckman Sychron CX5). |
| **Notes** | The average serum levels at the specified time intervals were extracted from the concentration-time curve of the two regimens. |

| **Chuan et al.^17^** | **Characteristics** |
| --- | --- |
| **Study design** | Retrospective review of hospital database and pharmacokinetic model. |
| **Study objective** | To determine the population pharmacokinetics of magnesium from sparse observational data in patients with pre-eclampsia. |
| **Participants** | 116 women with severe pre-eclampsia who had MgSO_4_. Severe pre-eclampsia was defined as blood pressure >160/110 mmHg, persistent severe headaches, visual disturbances, more than 2 clonus, elevated aspartate or alanine transferase levels to twice normal with epigastric or right upper-quadrant pain. The mean age of participants was 27 years and there were mainly white (99) and Asian (11). The weight of the participants during pregnancy was not provided.  Exclusion criteria: Not provided.  Setting: Mater Mother’s Hospital and the Royal Women’s Hospital, Brisbane, Queensland, Australia. |
| **Dosage regimen** | Participants had 4-5 g of MgSO_4_ over 15 minutes and maintenance infusion of 1 g per hour for 24 hours. |
| **Outcomes** | Pharmacokinetic profile of MgSO_4_ in women with severe pre-eclampsia using the NONMEM program in a 1-compartment model. NONMEM (version 5, level 1.1; University of California, San Francisco) on a personal computer in conjunction with the Microsoft PowerStation Fortran 77 compiler (version 1.00; Microsoft Corporation, Redmond, Wash). |
| **Laboratory method of estimating serum magnesium concentration** | Magnesium assay was determined by the dye-binding colorimetry in which magnesium ion formed a purple-red complex with xylidyl blue in alkaline solution and detected at 600 nm. |
| **Notes** | Pharmacokinetic profile was derived from the model. |

| **Cruikshank et al.^18^** | **Characteristics** |
| --- | --- |
| **Study design** | Cross-sectional study. |
| **Study objective** | To study the maternal urinary excretion of magnesium, calcium and phosphate in patients with intravenous magnesium sulfate. |
| **Participants** | 20 women in labour at term with mild to moderate pre-eclampsia. Mild or moderate pre-eclampsia was not defined. The demographic characteristics of the participants were not provided.  Exclusion criteria: Not stated  Setting: University of Iowa College of Medicine and University of Illinois at the Medical Centre, Chicago, Illinois, USA. |
| **Dosage regimen** | 4 g intravenous loading dose over 15-30 minutes followed by 1-2 g per hour maintenance dose. Infusion was continued up to 24 hour after delivery. |
| **Outcomes** | urinary magnesium, calcium and phosphate. |
| **Laboratory method of estimating serum magnesium concentration** | Urine samples were collected in aliquots from an in-dwelling catheter. Maternal blood was collected before and after the infusion. Magnesium determination in urine and blood were done by atomic absorption spectrophotometry (Perkin-Elmer model 303). |
| **Notes** | None. |

| **Dayicioglu et al.^19^** | **Characteristics** |
| --- | --- |
| **Study design** | Cross-sectional study. |
| **Study objective** | To determine if with standard dose of MgSO_4_ to pre-eclamptic women there is a significant change in serum magnesium levels according to body mass indices and if the changes affect the rate of preventing eclamptic seizures. |
| **Participants** | 194 women with pre-eclampsia. Hypertension was defined as blood pressure 140/90 mmHg, using Korotkoff phase V to define diastolic pressure. Proteinuria was described as 300 mg or more of proteinuria per 24 hours or persistent 30 mg/dl (1+dipstick) in random urine samples. Pre-eclampsia was considered severe in the presence of persistent high systolic (160mm Hg) and diastolic (110mm Hg) blood pressure despite antihypertensive therapy, persistent severe headache, visual disturbance, and elevated aspartate or alanine transferase levels with epigastric or right upper-quadrant pain.  Body mass index (BMI) was calculated using a standard formula (weight-kg/height-m^2^, Quetelet’s Index) based on body weight at the time of the study.  Exclusion criterion: Abnormal renal function suggested by laboratory results.  Setting: Zeynep Kamil Women and Children’s Disease Education and Research Hospital, Uskudar, Istanbul, Turkey |
| **Dosage regimen** | Intravenous 4.5 g loading dose of MgSO_4_ and 1.8 g/hour maintenance dose by an infusion pump. |
| **Outcomes** | Magnesium serum level correlation with body mass index of participants. |
| **Laboratory method of estimating serum magnesium concentration** | Method used to estimate serum magnesium was not reported. |
| **Notes** | Women with mild pre-eclampsia received MgSO_4_ in the intrapartum and postpartum periods. MgSO_4_ treatment was stopped after 24 hours. No additional dose of MgSO_4_ was given when sub-therapeutic levels were detected. |

| **Ekele and Badung ^20^** | **Characteristics** |
| --- | --- |
| **Study design** | Cross-sectional study. |
| **Study objective** | To estimate the serum levels of magnesium in eclamptic women on magnesium sulphate and relate serum levels with clinical findings. |
| **Participants** | 19 women who were admitted for eclampsia and had magnesium sulphate as the sole anticonvulsant. What constituted eclampsia was not reported. Modified Pritchard regimen, in which the 5 g intramuscular maintenance was stopped after 12 hours. The mean age and body mass index was 18.3 years and 21.9 kg/m^2^ respectively  Exclusion criteria: women who had Diazepam or any other anticonvulsant agent before or at admission.  Setting: Usman Danfodiyo University Teaching Hospital, Sokoto, Nigeria. |
| **Dosage regimen** | 14 g (4 g intravenous + 10 g intramuscular) loading dose of MgSO_4_ and intramuscular maintenance dose of 5 g 4 hourly for 12 hours. |
| **Outcomes** | Serum levels of magnesium in relation with clinical indicators. |
| **Laboratory method of estimating serum magnesium concentration** | Venous blood was collected before the loading and before the each of the maintenance doses. Unhaemolysed blood separated through centrifugation was assayed using Xylidyl blue colorimetric principle and Jenway 6051 colorimeter. |
| **Notes** | None. |

| **Guzin et al.^21^** | **Characteristics** |
| --- | --- |
| **Study design** | Cross-sectional study. |
| **Study objective** | To observe the effects of magnesium sulfate on various components of the coagulation system in women with pre-eclampsia. |
| **Participants** | 50 women: 5 with eclampsia and 45 with severe pre-eclampsia participated. Severe pre-eclampsia was defined as blood pressure ≥160/110 mmHg on two occasions, 6 hours apart or, if proteinuria was 5 g or more in 24 hours. Severity of pre-eclampsia was included features of end-organ damage and laboratory abnormalities of HELLP. Eclampsia was occurrence of convulsion in pre-eclamptic cases due to no other causes. The participants’ age and weight were not reported.  Exclusion criteria: women with history of bleeding disorders, hypertension, liver disease or recent use of antiplatelet medications.  Setting: Obstetrics Department of Goztepe Education and Research Hospital, Istanbul, Turkey. |
| **Dosage regimen** | 6 g intravenous loading dose over 20 minutes followed by 2 g/hour maintenance infusion until 12 hours postpartum. |
| **Outcomes** | Blood biochemical parameters and coagulation status. |
| **Laboratory method of estimating serum magnesium concentration** | Not reported. |
| **Notes** | Average serum magnesium of eclamptic and pre-eclamptic women was reported. |

| **Handwerker et al.^22^** | **Characteristics** |
| --- | --- |
| **Study design** | Cross-sectional study. |
| **Study objective** | To demonstrate the levels of magnesium ion change during standard intravenous MgSO_4_ therapy. |
| **Participants** | 8 women randomly selected following a diagnosis of pre-eclampsia defined by elevated blood pressure ≥ 140/90 mmHg and at least 1+ proteinuria by semi-quantitative assay. The women did not eat/drink in labour but had Ringer’s Lactate solution intravenously at 125 ml per hour which was reduced to 75 ml/hour when MgSO_4_ was started. The mean participants’ age was 26.9 years and mean gestational age was 39.2 weeks. No information on maternal weight was provided.  Exclusion criteria: Not stated  Setting: Department of Obstetrics and Gynaecology, The New York Hospital Medical Center of Queens, New York, USA. |
| **Dosage regimen** | 4 g intravenous loading dose infusion over 30 minutes followed by 2 g/hour maintenance dose. |
| **Outcomes** | Ionized serum magnesium level. |
| **Laboratory method of estimating serum magnesium concentration** | Ionized and total serum magnesium were analyzed with NOVA Biomedical Stat Profile 8 Analyzer and Kodak Ektachem DT-60 Analyzer respectively. |
| **Notes** | None. |

| **Lu et al.^23^** | **Characteristics** |
| --- | --- |
| **Study design** | Cross-sectional study and pharmacokinetic-pharmacodynamic model. |
| **Study objective** | To describe the relationship between plasma magnesium concentration and blood pressure response in pregnant women with pre-eclampsia. |
| **Participants** | 51 hospitalized women who received MgSO_4_ as their only treatment for pregnancy-induced hypertension. Gestational hypertension was defined as systolic blood pressure of at least 140 mmHg with a > 30 mmHg rise and/or diastolic blood pressure of at least 90 mmHg with a rise of >15nnHg occurring on two or more occasions after 20 weeks of gestation. Proteinuria was defined as a urine protein concentration of at least “2+”. Participants’ demographic data was not provided  Exclusion criteria: Pre-existing hypertension or cardiac or renal disease. The use of anti-hypertensive, vasodilators and vasoconstrictors (theophylline, tricyclic antidepressants, antipsychotic and anti-inflammatory drugs) were additional exclusion criteria.  Setting: Department of Obstetrics and Gynecology, Jinling Hospital, Nanjing, China. |
| **Dosage regimen** | Intravenous MgSO_4_ 120 mg/kg loading dose over 1 hour followed by maintenance dose of 24 mg/kg for 5 hours. |
| **Outcomes** | Relationship between plasma magnesium concentration and blood pressure response. |
| **Laboratory method of estimating serum magnesium concentration** | The method of estimation of serum magnesium prior to fitting the data into the model was not provided. A 2-step strategy of pharmacokinetic-pharmacodynamic model was used to fit data into NONMEM program. |
| **Notes** | None. |

| **Manorot et al.^24^** | **Characteristics** |
| --- | --- |
| **Study design** | Randomised control trial. |
| **Study objective** | To compare serum level between intravenous and intramuscular maintenance regimen in women with severe pre-eclampsia |
| **Participants** | 50 women admitted into the antenatal ward or labor room on account of severe pre-eclampsia with singleton pregnancy. The mean age, weight and gestational age of the women was 26.98 years, 65.25 kg and 36.3 weeks respectively. The diagnostic criteria for severe pre-eclampsia were not stated.  Exclusion criteria: Presence of other medical or surgical complications or contraindication to MgSO_4_ use.  Setting: Department of Obstetrics and Gynecology, Maharaj Nakorn Chiang Mai Hospital, Chiang Mai, Thailand. |
| **Dosage regimen** | Zuspan (4 g IV loading dose + 1 g/ hour maintenance continuous infusion) or Pritchard (14 g; 4 g IV + 10 IM loading dose followed by 5 g IM maintenance dose every 4 hours) regimen. |
| **Outcomes** | Serum magnesium level between intravenous and intramuscular maintenance regimens. |
| **Laboratory method of estimating serum magnesium concentration** | All samples were frozen till the end of the study. Serum magnesium was determined by methylthymol blue method with the use of spectrophotometry |
| **Notes** | None. |

| **Mason et al.^25^** | **Characteristics** |
| --- | --- |
| **Study design** | Case-control study. |
| **Study objective** | To determine the effects of magnesium sulphate therapy on ionized magnesium in the cord blood of pregnancies complicated by pre-eclampsia. |
| **Participants** | 37 pregnant women at ≥ 24weeks gestation with an indication for MgSO_4_ for either preterm labour or pre-eclampsia. 93% of the women were black. No information was provided on inclusion criteria and demographic characteristics of the women.  Exclusion criteria: not stated  Setting: Hutzel Hospital, Detroit, USA |
| **Dosage regimen** | 6 g intravenous loading dose of MgSO_4_ and 2 g/hour continuous infusion maintenance dose. |
| **Outcomes** | Correlation of fetal ionized magnesium levels with maternal ionized magnesium. |
| **Laboratory method of estimating serum magnesium concentration** | Maternal blood samples and fetal umbilical vein blood samples were collected anaerobically, immediately after delivery. Derived serum was stored at -70 degree Celsius. Ionized and total magnesium was analyzed with Nova Biomedical Stat Profile 8 and Nova Nucleus Biomedical Stat Profile analyzers respectively (Nova Biomedical, Waltham,Mass.). |
| **Notes** | Data on women with pre-eclampsia only. |

| **Phuapradit et al.^26^** | **Characteristics** |
| --- | --- |
| **Study design** | Cross-sectional study. |
| **Study objective** | To review the use of intravenous magnesium sulphate in pre-eclampsia. |
| **Participants** | 44 women with severe pre-eclampsia attending the antenatal clinic. The definition of severe pre-eclampsia was not provided. The women had a mean age, weight and were at a mean gestational age of 26 years, 70.4 kg and 35.4 weeks respectively.  Exclusion criteria: Not stated  Setting: Department of Obstetrics and Gynaecology, Ramathibodi Hospital, Mahidol University, Bangkok, Thailand. |
| **Dosage regimen** | 5 g intravenous loading dose of MgSO_4_ over 15 minutes followed by 1 g/hour intravenous infusion until 24 hours postpartum. |
| **Outcomes** | Serial serum levels of magnesium. |
| **Laboratory method of estimating serum magnesium concentration** | Serum magnesium was analysed by atomic absorption spectrophotometry either immediately or from frozen samples previously stored for up to 48 hours. |
| **Notes** | None. |

| **Salinger et al.^27^** | **Characteristics** |
| --- | --- |
| **Study design** | Randomised control trial and population pharmacokinetic model. |
| **Study objective** | To compare magnesium sulphate concentrations achieved by intramuscular and intravenous regimens used for the prevention of eclampsia. |
| **Participants** | 258 women with pre-eclampsia who participated in a randomized trial that compared two methods of MgSO_4_ administration. The definition of pre-eclampsia used was not provided. The women had a mean age, weight and were at a mean gestational age of 24.5 years, 56.4kg and 33.9 weeks respectively.  Exclusion criteria: Treatment interruption before blood sampling, failure to collect blood sample and mislabeled blood sample.  Setting: Government Medical College (GMC), Nagpur and Christian Medical College (CMC), Vellore, India. |
| **Dosage regimen** | Zuspan regimen of 4 g intravenous infusion loading dose followed by 1 g/hour maintenance dose (Springfusor® pump, Go Medical, Subiaco, Australia), and Pritchard regimen of 14 g (IV 4 g + IM 10 g) loading dose followed by 5 g every 4 hours intramuscular maintenance dose. |
| **Outcomes** | Pharmacological equivalence of the two regimens and an estimate of intramuscular bioavailability of magnesium. |
| **Laboratory method of estimating serum magnesium concentration** | Blood was collected at specified intervals and analyzed on-site. Analysis was done with Selectra E (Merck Chemicals, Mumbai, India) at GMC-Nagpur and with Autopure Magnesium (Hitachi, Roche Diagnostics India, Mumbai, India) at CMC-Vallore. |
| **Notes** | Outcome measures were reported without separating the data from each of the regimens. |

| **Seydoux et al.^28^** | **Characteristics** |
| --- | --- |
| **Study design** | Cross-sectional study. |
| **Study objective** | To determine serum and lymphocyte magnesium concentrations during normal pregnancy and to compare the magnesium status in the third trimester of pregnancy between women with normal pregnancy, and those with gestational hypertension or pre-eclampsia. |
| **Participants** | 5 women with severe pre-eclampsia who had MgSO_4_. Pre-eclampsia was defined as hypertension and proteinuria ≥ 300mg/24 hour or >1 g/l in random urine samples. The demographic characteristics of the women were not separated from those of women who did not use MgSO_4_.  Exclusion criteria: Women with essential hypertension, those with prior Magnesium or diuretic treatment, and those with history of renal disease.  Setting: Department of Obstetrics and Gynaecology, University Hospital of Geneva, Switzerland. |
| **Dosage regimen** | 2 g intravenous and 10 g intramuscular (12 g) loading dose of MgSO_4_. No maintenance dose was administered. |
| **Outcomes** | Serum and intralymphocytic magnesium concentration and urinary magnesium excretion. |
| **Laboratory method of estimating serum magnesium concentration** | Plasma magnesium was determined by atomic absorption spectrophotometry. Intracellular Magnesium concentration was measured in lymphocytes as described by Elin & Johnson (1985). |
| **Notes** | Data was extracted for only women who had MgSO_4_ infusion. |

| **Shreya et al.^29^** | **Characteristics** |
| --- | --- |
| **Study design** | Comparative study. |
| **Study objective** | To study efficacy of Single dose of MgSO_4_ and Pritchard regimen in treatment of imminent eclampsia and eclampsia and to correlate clinical and biochemical parameters of serum magnesium in both regimens. |
| **Participants** | 27 women with eclampsia (defined as convulsions in a woman with pre-eclampsia that cannot be attributed to other causes) and 53 women with imminent eclampsia (who had any one of the following symptoms: persistent headache, visual disturbances and epigastric pain) were recruited in this study. The demographic characteristics of the women with eclampsia and imminent eclampsia were not reported separately. Overall, 58% of women were between 21-29 years, 71% were at gestation age 28-36 weeks and 56% were primigravida.  Exclusion criteria: Women were not willing to participate, those received any anticonvulsant or MgSO_4_ before coming to the study hospital, and those having contraindications for MgSO_4_  Setting: People Education Society’s Institute of Medical Sciences and Research Hospital, Kuppam, India |
| **Dosage regimen** | Pritchard regimen: 4 g IV and 10 g IM loading dose of MgSO_4_ followed by 5 g per 4 hours IM maintenance.  Single dose: 4 g IV followed by 4 g IM of MgSO_4_. No maintenance dose was given. |
| **Outcomes** | Serum magnesium level in women with eclampsia treated by Pritchard and single dose regimens. |
| **Laboratory method of estimating serum magnesium concentration** | Method of estimation of serum magnesium was not reported. |
| **Notes** | The average serum levels at the specified time intervals were extracted of the two regimens. |

| **Sibai et al.^5^** | **Characteristics** |
| --- | --- |
| **Study design** | Cross-sectional study. |
| **Study objective** | To compare the magnesium levels obtained in the treatment of pre-eclamptic patients with the intramuscular regimen of Pritchard to the levels in the continuous intravenous regimens with maintenance doses of 1 gm/hr and 2 gm/hr. |
| **Participants** | 32 women with pre-eclampsia: 10 with mild and 22 with severe cases of pre-eclampsia. Mild pre-eclampsia was defined as blood pressure 140/90 mmHg with proteinuria. Severe pre-eclampsia was defined as blood pressure of at least 160/110mmHg (on two occasions, 6 hours apart) with proteinuria. The range of the women’s age, weight and gestational age was 16-25 years, 119-199 kg and 34-40 weeks respectively.  Exclusion criterion: Not stated.  Setting: E. H. Crump Women’s Hospital and Perinatal Center, Memphis, Tennessee, USA. |
| **Dosage regimen** | Intravenous 4 g loading dose of magnesium sulphate and either 1 g or 2 g per hour continuous infusion by an infusion pump in one group. In the other group Pritchard regimen was administered to women with severe pre-eclampsia. Women with mild pre-eclampsia had only 10 g IM loading dose MgSO_4_ and 5 g every 4 hours IM maintenance dose. |
| **Outcomes** | Serum magnesium level in pre-eclampsia in women treated with different regimens. |
| **Laboratory method of estimating serum magnesium concentration** | Atomic absorption spectrophotometry was used to estimate serum magnesium immediately or from frozen sample stored up to 48 hours after sample collection. |
| **Notes** | Data for women with mild and severe pre-eclampsia was presented together. |

| **Singh et al.^30^** | **Characteristics** |
| --- | --- |
| **Study design** | Cross-sectional study. |
| **Study objective** | To estimate the serum magnesium levels in pre-eclampsia and eclampsia and to study the effect of using different regimens of magnesium sulphate. |
| **Participants** | 70 women participated in the study: 35 women with pre-eclampsia and 35 women with eclampsia. Pre-eclampsia was defined as blood pressure 140/90 mmHg or more, proteinuria and oedema with/without history of convulsions in the last trimester of pregnancy, during labour or within 48 hours of delivery. The mean age of the women was 23.6 years. No information on their weight was provided and the reported gestational age included those of normal women who had no pre-eclampsia.  Exclusion criterion: Pre-existing hypertension, renal disease, diabetes mellitus, thyrotoxicosis or other secondary causes of hypertension, hydatidiform mole.  Setting: Department of Pharmacology and Obstetrics and Gynaecology of S. N. Medical College and Hospital, Agra, Uttar Pradesh, India. |
| **Dosage regimen** | 1. IM loading dose of 5 g MgSO_4_ followed by 4 g IV doses for eclampsia.  2. IV 6 g MgSO_4_ loading dose, followed by IV continuous maintenance dose of 2 g/hour. |
| **Outcomes** | Serum magnesium levels. |
| **Laboratory method of estimating serum magnesium concentration** | Atomic absorption spectrophotometer (Model AAS-4139) was used to measure the serum magnesium. |
| **Notes** | Serum concentration of magnesium was extracted from a concentration-time curve. In addition, the data was presented based on the regimen the women received and thus the proportion of those with pre-eclampsia or eclampsia is unknown. |

| **Suvarna et al.^31^** | **Characteristics** |
| --- | --- |
| **Study design** | Cross-sectional study. |
| **Study objective** | Not reported |
| **Participants** | 126 women with pre-eclampsia (53) or eclampsia (73).The inclusion criteria were not reported. The demographic characteristics of the women include a mean age, body weight and gestational age of 24.6 years, 55.2 kg and 33.5 weeks respectively.  Exclusion criteria: Not stated  Setting: Kasturba Medical College and Government Lady Goshen Hospitals, Mangalore, India. |
| **Dosage regimen** | 14 g (4 g IV + 10 g IM) loading dose and 5 g 4 hourly intramuscular maintenance dose compared with 4 g intravenous loading dose over 15-20 minutes followed by IM/IV 2 g maintenance dose every 3 hours. |
| **Outcomes** | Serum levels of magnesium with Pritchard regimen and a low dose regimen of MgSO_4_. |
| **Laboratory method of estimating serum magnesium concentration** | Method of estimation of serum magnesium was not provided. |
| **Notes** | None. |

| **Taber et al.^32^** | **Characteristics** |
| --- | --- |
| **Study design** | Case-control study and pharmacokinetic model |
| **Study objective** | To determine the correlation between ionized magnesium and total magnesium under basal and therapeutic conditions and to define the initiation and elimination pharmacokinetics of both forms during intravenous MgSO_4_ infusion. |
| **Participants** | 9 women > 24 weeks pregnant with pre-eclampsia who had an indication for magnesium sulphate. What constitutes pre-eclampsia used to recruit women was not defined. The women were mostly Hispanic (89%) with age range of 18-35 years. They had a mean gestational age and body mass index of 37.5 weeks and 36.9 kg/m^2^ respectively.  Exclusion criteria: Patients with previous magnesium administration during the pregnancy, diabetes mellitus or other active endocrinopathies, or preexisting renal disease.  Setting: University Medical Center, California, USA. |
| **Dosage regimen** | 4 g loading intravenous dose of MgSO_4_, followed by 2 g/hour maintenance dose. |
| **Outcomes** | Serum total magnesium and ionized magnesium. Initiation and elimination pharmacokinetics of MgSO_4_ during intravenous infusion. |
| **Laboratory method of estimating serum magnesium concentration** | Ionized magnesium was analyzed using ion-selective electrodes from whole blood with an ion-selective electrode (NOVA 8 Analyzer; NOVA Biomedical Corp, Waltham, Mass). Samples for total magnesium was prepared by centrifugation for 10 min at 2500 revolutions/min and serum magnesium analysis was done by a xylidyl blue spectrophotometric method from blood samples frozen at – 20 degree Celsius. |
| **Notes** | None. |

| **Tongsong et al.^33^** | **Characteristics** |
| --- | --- |
| **Study design** | Randomized controlled trial. |
| **Study objective** | To compare the maintenance dose of MgSO_4_ 1g versus 2g per hour in terms of:   1. rate of achieving therapeutic level (4-7 mEq/L) of serum Mg 2. mean serum magnesium level at 2 and 4 hours after loading dose and 2 hours after delivery. |
| **Participants** | Pregnant women with severe pre-eclampsia or eclampsia |
| **Dosage regimen** | 1 g versus 2 g / hour of MgSO_4_ IV infusion after a loading dose of 4g IV. |
| **Outcomes** | 1. serum magnesium level mEq/L at 2 and 4 hours after the initial loading dose and 2 hours after delivery 2. rate of achieving therapeutic level (4-7 mEq/L) of serum magnesium at 2 and 4 hours after the initial loading dose and 2 hours after delivery |
| **Laboratory method of estimating serum magnesium concentration** | Methylthymol blue using spectrophotometry by the same person who was blinded to the dose of MgSO_4_ |
| **Notes** | None. |

| **Thurnau et al.^34^** | **Characteristics** |
| --- | --- |
| **Study design** | Case-control study. |
| **Study objective** | To correlate serum magnesium levels with those of cerebrospinal fluid in patients with pre-eclampsia receiving intravenous MgSO_4_ and determine whether the magnesium ion crosses the blood- brain barrier in significant amount. |
| **Participants** | 10 women with pre-eclampsia who had MgSO_4_ at least 4 hours (range 4 ½ -72 hours) prior to delivery. Pritchard’s definition was referenced and used to recruit women. Individual participant’s age and gestational age were presented, but maternal weight was unreported.  Exclusion criterion was a contraindication to spinal anaesthesia.  Setting: University of Oklahoma College of Medicine, USA. |
| **Dosage regimen** | 6 g loading dose of MgSO_4_ followed by 2 g/hour IV maintenance dose. |
| **Outcomes** | Cerebrospinal fluid and serum magnesium level. |
| **Laboratory method of estimating serum magnesium concentration** | Lumbar puncture was performed with a 25 gauge 3 ½ inch needle and 1 ml of cerebro-spinal fluid collected. 3 ml of blood was collected simultaneously by venipuncture.  Assays of cerebro-spinal and serum magnesium were done with automatic clinical analyzers (a modification of the methylthymol blue complexometric procedure). |
| **Notes** | None. |

| **Wright et al.^35^** | **Characteristics** |
| --- | --- |
| **Study design** | Cross-sectional study. |
| **Study objective** | To measure the apparent volume of distribution (AVOD) of magnesium in pre-eclampsia and preterm labour and determine if a standard 4 gm loading dose of MgSO_4_ will attain therapeutic levels. |
| **Participants** | 25 consecutive women with pre-eclampsia. Pre-eclampsia was defined as blood pressure >140/90mmHg and 1+ proteinuria in catheterized urine specimen. The age of the women was not reported but their mean weight and gestational age was 80.1 kg and 38.8 weeks respectively.  Exclusion criteria: Not stated.  Setting: University of Texas Health Center, Houston, USA. |
| **Dosage regimen** | Intravenous 4 g infusion of magnesium sulphate over 15 minutes. |
| **Outcomes** | Serum level of magnesium and apparent volume of distribution. |
| **Laboratory method of estimating serum magnesium concentration** | Blood was drawn from separate sites immediately before and 1 minute after administration of MgSO_4_. Magnesium levels were determined using Calmagite reaction and a Paramax System (Baxter Scientific, Irvine, CA). |
| **Notes** | Correction was made for initial volume of distribution since women in labour received significant volumes of intravenous fluid. |

| **Yoshida et al.^36^** | **Characteristics** |
| --- | --- |
| **Study design** | Cross-sectional study. |
| **Study objective** | To estimate the relationship between ionized and total Mg levels during MgSO_4_ administration in women with preterm labour and pre-eclampsia. |
| **Participants** | Sixteen women with pre-eclampsia, defined as blood pressure was ≥140/90 mmHg plus proteinuria or oedema that is generalized and overt or both at about 33 weeks gestation. The mean gestational age of their pregnancy was 33.4 weeks. The age and weight of the women were not reported.  Exclusion criteria: pregnancies with pre-existent renal disease or active endocrine disorders were excluded.  Setting: Tokyo Women Medical University Hospital, Japan |
| **Dosage regimen** | Intravenous magnesium sulphate 4 g loading dose and 1-2 g/hr maintenance dose. The total dose of MgSO_4_ for pre-eclampsia was 100 g. |
| **Outcomes** | The relations between ionized and total Mg levels. |
| **Laboratory method of estimating serum magnesium concentration** | Selective ion electrode (NOVA 8 Analyzer, NOVA Biomedical Corp, Waltham, MA) was used to measure ionized magnesium at bedside while total serum magnesium was measured in the laboratory with a Hitachi 7700 automatic analyzer. |
| **Notes** | Data extraction was only for the 16 women treated for pre-eclampsia. |
