# Supplementary material for: Clinical pharmacokinetic properties of magnesium sulphate in women with pre‐eclampsia and eclampsia
Source: BJOG. 2015 Nov 24;123(3):356–66. doi: 10.1111/1471-0528.13753 (PMC4737322; doi:10.1111/1471-0528.13753)
Supplement: Supplementary file 6 — Table S3. Characteristics of excluded studies and reasons for exclusion. [file BJO-123-356-s006.pdf]

Table S3. Characteristics of excluded studies and reasons for exclusion

Aali 2007b	Characteristics
**Study design**	Cross-sectional study
**Study objective**	To determine the alterations of serum ionized magnesium and calcium and their relationship in patients with severe preeclampsia/eclampsia.
**Participants**	46 women with severe preeclampsia and 4 women with eclampsia who had MgSO4. Severe preeclampsia was defined as blood pressure ≥160/110mmHg and proteinuria ≥2+. Eclampsia was taken as convulsion in a woman with preeclampsia not attributable to other causes.<br>Exclusion criteria: Not stated<br>Setting: Maternity center of Afzalipour Hospital, Kerman City, South-East Iran.
**Dosage regimen**	4 gram intravenous loading dose and 2 gram continuous intravenous maintenance dose of MgSO4.
**Outcomes**	Serum ionized magnesium and calcium.
**Laboratory method of estimating serum magnesium concentration**	Serum from venous blood collected was kept at -20 degree. Total magnesium analyzed with an atomic absorption spectroscope while ionized magnesium was analyzed by dye-binding colorimetry
**Reasons for exclusion**	Data of ionized magnesium is a replica of the results of ionized magnesium of the included study of the same author published in the same year (Aali 2007a)

Apostol 2009	Characteristics
**Study design**	Cross-sectional study.
**Study objective**	To study the distribution of ionized and total magnesium in serum and cerebral spinal fluid (CSF) and effect of treatment on calcium and ionized Ca:Mg ratios.
**Participants**	32 women; 16 women healthy pregnant women and 16 women with preeclampsia.<br>Exclusion criteria: These were history of neurologic disease, renal disease, hypertension, vascular disease and preterm labour.<br>Setting: Department of Obstetrics and Gynecology, Downstate Medical Center, Brooklyn, New York, USA.
**Dosage regimen**	Intravenous 6 gram loading dose over 15-20 minutes followed by 2 gram/ hour maintenance dose.
**Outcomes**	Ionized and total magnesium in serum and cerebral spinal fluid.
**Laboratory method of estimating serum magnesium concentration**	Not reported
**Reasons for exclusion**	This was an abstract and a duplication of results of an included study- Apostol 2010.

Brookfield 2015	Characteristics
**Study design**	Prospective study from 10/2012 to 5/2014
**Study objective**	To characterize the PK of MgSO4 in pregnant women so that optimal, PK/PD-derived dosing schedules can be determined for women receiving MgSO4 for obstetric indications.
**Participants**	151 pregnant women with preeclampsia, preterm labor or prematurity (< 32 weeks) who consented to participate and prescribed MgSO4 were recruited.<br>Exclusion criteria: Not stated.<br>Setting: Not stated.
**Dosage regimen**	Intravenous 4 gram loading dose and 2 gram per hour maintenance dose of MgSO4.
**Outcomes**	Serum MgSO4 concentration at baseline, 30 min, 1 h, 2 h, 4 h, and q6 h during MgSO4 infusion; and at 1 h, 3 h, 6 h, 9 h, and 12 h after MgSO4 was discontinued. A two-compartment linear disposition model was used to describe PK data with nonlinear mixed-effects modeling and visual predictive check for estimated modeling of observed and predicted serum MgSO4 concentrations.
**Laboratory method of estimating serum magnesium concentration**	Method used to measure serum magnesium was not reported.
**Reasons for exclusion**	Only published conference abstract is currently available. Full paper is presently being prepared according to the authors. The study currently does not have data disaggregated between women with pre-eclampsia and preterm labour.

Boriboonhirunsarn 2012	Characteristics
**Study design**	Cross-sectional study
**Study objective**	To determine the correlation between cord blood and maternal serum magnesium levels among pre-eclamptic pregnant women treated with magnesium sulfate.
**Participants**	36 pregnant women with preeclampsia at >28 weeks gestation. Participants had a mean maternal age of 27.4 years, body mass index of 27.5kg/m^2 and were at a mean gestational age of 38 weeks. Preeclampsia was defined as blood pressure >140/90 mmHg and proteinuria of ≥30mg/dl (≥1+ reading on dipstick) in a random urine.<br>Exclusion criteria: Pregnancy <28 weeks gestation, multifetal pregnancy, renal insufficiency and history of use of drugs that could interact with magnesium or calcium metabolism.<br>Setting: Department of Obstetrics and Gynecology, Faculty of Medicine Siriraj Hospital, Mahidol University, Bangkok, Thailand.
**Dosage regimen**	4 gram intravenous loading dose followed by 2 gram/hour intravenous infusion for an average of 5.1 hours prior to delivery.
**Outcomes**	Cord blood and maternal serum magnesium.

**Laboratory method of estimating serum magnesium concentration**	Blood was collected immediately after delivery in lithium heparin tubes and magnesium levels were estimated using colorimetric end-points.
**Reasons for exclusion**	Dosage adjustments were performed as indicated depending on if the maternal serum magnesium level was within therapeutic range and based on clinical signs and symptoms.

**Chao 1990**	**Characteristics**
**Study design**	Cross-sectional study
**Study objective**	To quantitate the changes in patellar reflex response accompanying transition from sub-therapeutic to therapeutic serum magnesium levels in preeclamptic women.
**Participants**	20 women admitted for labour and delivery<br>Exclusion criteria: Women with metabolic or endocrine disorders with associated spasticity or hyporeflexia. Those who had epidural were also excluded.<br>Setting: Department of Obstetrics and Gynecology, University of California at Los Angeles Medical Center, Los Angeles, USA.
**Dosage regimen**	Intravenous 4 gram MgSO4 over 20 minutes followed by 2 gram/ hour maintenance dose.
**Outcomes**	Patellar reflex and serum magnesium level.
**Laboratory method of estimating serum magnesium concentration**	Atomic absorption spectrophotometer (Varian AA 1475, Varian Instrument Division, Palo Alto, CA).
**Reasons for exclusion**	Testing for tendon reflexes were deferred in women in second stage and during episodes of fetal heart rate abnormalities.

**Charoenvidhya 2013**	**Characteristics**
**Study design**	Cross-sectional study
**Study objective**	The percentage of women who reached the therapeutic serum magnesium level of 2-3.5mmol/L in the two groups
**Participants**	60 women with preeclampsia who were either given 5gram intravenous loading and 2 gram/hour maintenance dose (30 women) or 5gram intravenous loading dose and 1/hour loading dose (30 women).<br>Exclusion criteria: Not stated<br>Setting: King Chulalongkorn Memorial Hospital (KCMH), Bangkok, Thailand.
**Dosage regimen**	5 gram intravenous loading dose of MgSO4 and either 2 gram/hour or 1 gram/hour intravenous maintenance dose.
**Outcomes**	Serum magnesium level of 2-3.5mmol/L in the two groups.
**Laboratory method of estimating serum magnesium concentration**	Blood samples were taken in tubes and analyzed by magnesium kit (Cobas®).

**Reasons for exclusion**	The results were presented as the number (percentage) of participants who attained therapeutic levels. Mean of serum levels was not reported.

**Chen 1991b**	**Characteristics**
**Study design**	Open label RCT study design.
**Study objective**	To develop population PK and PKPD (blood pressure) models for MgSO4 in women with pregnancy induced hypertension.
**Participants**	10 participants with pregnancy induced hypertension (Criterion not reported). Most participants were young, mean age of 28.1 (SD=3.31) years (range 24-36). Exclusion criteria: Not stated<br>Setting: General Hospital of Nanjing Armed Forces, Nanjing, P.R. China.
**Dosage regimen**	Fast IV infusion 7.5 g in 1h followed by slow IV infusion 7.5 g in 5h.
**Outcomes**	Serum magnesium (mmol/L). Mean/SD at 1, 6, 7, 9, 11 and 15 post IV infusion (reported as observed value-baseline).<br><br>Population 2 compartment PK model: alpha, beta, K21 and Vc parameters estimates.<br><br>Population Emax effect compartment PD model: Emax,Ce50, kle and Keo estimates.
**Laboratory method of estimating serum magnesium concentration**	Magnesium levels were estimated using colorimetric end-points at selected time points. Linear range (12-72 ug/ml). Precision CV%<2%. Recovery% 100.6+/-1.7%. LOQ=1 ug/ml.
**Reasons for exclusion**	Study participants were women with pregnancy-induced hypertension.

**Cruikshank 1982**	**Characteristics**
**Study design**	Case control study
**Study objective**	To determine the effect of intrapartum magnesium sulphate therapy on the mineral content of colostrum.
**Participants**	10 women with preeclampsia who had been in labour between 37 and 40 weeks gestation. What constituted preeclampsia was not defined and maternal age and weight were not provided. None of the women breastfed at time of recruitment.<br>Exclusion criteria: Use of a diuretic.<br>Setting: University of Iowa College of Medicine, USA.
**Dosage regimen**	4 gram intravenous loading dose of MgSO4 followed by 1 gram per hour maintenance dose infusion up to 24 hours after delivery.
**Outcomes**	Mineral content of colostrum.
**Laboratory method of estimating serum magnesium concentration**	Breast milk was collected by manual expression or breast pump. Magnesium determination in milk and serum by atomic absorption spectrophotometry (Perkin-Elmer Model 303).
**Reasons for exclusion**	Serum magnesium was estimated “24 hours post-partum”

Flowers 1962	Characteristics
**Study design**	Cross-sectional
**Study objective**	To determine a safe dosage schedule based upon body weight and the volume of urinary excretion.
**Participants**	Women with severe preeclampsia and eclampsia.<br>Exclusion criteria: Not stated<br>Setting: University of North Carolina School of Medicine, Chapel Hill, N.C
**Dosage regimen**	Administration of MgSO4 based on body weight and the morning (8a.m) serum level of magnesium and the urinary excretion of magnesium. (Variable loading and maintenance doses). Intramuscular and intravenous routes utilized in the study.
**Outcomes**	Serum and urinary magnesium.
**Laboratory method of estimating serum magnesium concentration**	Estimation of serum magnesium and urinary excretion of magnesium used titan yellow method, assessed visually and compared (validated) with Beckman spectrophotometry.
**Reasons for exclusion**	Results were presented, in part, as a concentration-time curve for selected participants.<br>Determination of magnesium distribution was in animals (dogs).

Flowers 1965	Characteristics
**Study design**	Cross-sectional
**Study objective**	To determine a safe dosage schedule based upon body weight and the volume of urinary excretion.
**Participants**	Women with severe preeclampsia and eclampsia.<br>Exclusion criteria: Not stated<br>Setting: University of North Carolina School of Medicine, Chapel Hill, N.C.
**Dosage regimen**	Administration of MgSO4 based on body weight and the morning (8a.m) serum level of magnesium and the urinary excretion of magnesium. (Variable loading and maintenance doses). Intramuscular and intravenous routes utilized in the study.
**Outcomes**	Serum magnesium and urinary excretion of magnesium.
**Laboratory method of estimating serum magnesium concentration**	Estimation of serum magnesium and urinary excretion of magnesium used titan yellow method, assessed visually when compared the known standards against a white background and are read as milligrams per cent
**Reasons for exclusion**	Results of 16 women (9 preeclamptic and 7 eclamptic) were presented as individual patient’s lowest and highest 24- hours serum levels of magnesium.

Fong 1995	Characteristics
**Study design**	Case- control study
**Study objective**	To determine the cerebrospinal fluid magnesium levels in patients with preeclampsia before treatment with $MgSO_4$ for comparison with those of normal gravidas and correlate CSF magnesium levels of preeclamptic women with serum levels in pre-treatment state.
**Participants**	20 women with mild preeclampsia. Mild preeclampsia was defined as blood pressure ≥140/90 mmHg, presence of edema and proteinuria ≥2+.The participants had a mean age and weight of 31.05 years and 81.34kg respectively and were at a mean gestational age of 36.6 weeks.<br>Exclusion criteria: Previous history of neurologic or renal disease and contraindication to spinal anaesthesia.<br>Setting: Department of Obstetrics and Gynecology, New York Hospital-Cornell Medical Center, New York, USA>
**Dosage regimen**	None
**Outcomes**	To determine if baseline cerebrospinal fluid magnesium levels in preeclamptic differs from those in normal pregnancy and if cerebrospinal fluid magnesium of women with preeclampsia correlate with serum levels.
**Laboratory method of estimating serum magnesium concentration**	Serum magnesium was estimated using the spectrophotometric assay using the calmagite complexometric measurements with the automatic clinical analyzer Beckman Synchron CX5/CX7 (Brea, CA).
**Reasons for exclusion**	Baseline data was retrieved for only the 20 women with preeclampsia

Ghahiri 2005	Characteristics
**Study design**	Cross-sectional study
**Study objective**	Determination of serum magnesium level after oral administration of magnesium and to compare its result with intravenous administration.
**Participants**	66 women with mild preeclampsia. Preeclampsia was defined as blood pressure >140/90 mmHg after 20th week of gestation and proteinuria > 300mg/24 hours. Preeclampsia was mild when headaches, epigastric pain, blurred vision, abnormal renal or liver function tests were not detected.<br>Exclusion criteria: Not stated.<br>Setting: Al-Zahra and Shahid Beheshti Hospitals, Isfahan, Iran.
**Dosage regimen**	Intravenous 2 gram/hour MgSO4 and oral magnesium chloride 4 gram/ 2 hours were compared.
**Outcomes**	Serum magnesium level.
**Laboratory method of estimating serum magnesium concentration**	Serum magnesium levels were assessed with colorimetric method
**Reasons for exclusion**	The mean serum level of magnesium was presented in a Concentration-Time Curve. Authors did not specify if mg/dl, mmol/L or mEq/L was the unit of measurement used.

Hallak 1993	Characteristics
**Study design**	Cross-sectional study
**Study objective**	To determine the effect of maternal intravenous magnesium sulfate administration on fetal serum and amniotic fluid magnesium.
**Participants**	36 women who had MgSO4 prior to invasive fetal blood sampling for prenatal diagnosis.<br>Exclusion criteria: Hydrops fetalis or hydropic placenta<br>Setting: Center for fetal Diagnosis and Therapy. Department of Obstetrics and Gynecology, Wayne State University/ Hutzel Hospital, Detroit, Michigan, USA.
**Dosage regimen**	6 gram intravenous loading dose over 20 minutes followed by 2 grams per hour maintenance infusion.
**Outcomes**	To determine the effect of maternal intravenous magnesium sulfate administration on fetal serum and amniotic fluid magnesium.
**Laboratory method of estimating serum magnesium concentration**	"Magnesium was analysed colorimetrically at 360nm"
**Reasons for exclusion**	$MgSO_4$ was administered for treatment of preterm labour.

Huang 1998	Characteristics
**Study design**	Open label study design
**Study objective**	To study the changes of Ca and Mg levels in patients with pregnancy induced hypertension and the therapeutic effect of $MgSO_4$.
**Participants**	14 young women with no pregnancy, 14 young pregnant women with no complications, and 36 participants with pregnancy induced hypertension (PIH) (mild, 14; moderate 10, severe 12). Most participants were young, range 18-25 years old, first pregnancy, no pre-pregnancy complications such as high blood pressure or renal impairment.<br>Exclusion criteria: Not stated<br>Setting: General Hospital of Nanjing Armed Forces, Nanjing, P.R. China.
**Dosage regimen**	IV infusion 1.5g/h x 4h.
**Outcomes**	Magnesium (mmol/L) levels in plasma and RBC.<br>- No pregnancy vs normal pregnancy vs pregnancy induced hypertension (mild, moderate, severe)<br>- Before and after MgSO4 treatment in pregnant women with PIH.
**Laboratory method of estimating serum magnesium concentration**	Magnesium levels in heparin-plasma and RBC (collected by sequential centrifugations) were estimated with an atomic absorption spectroscope (Hitachi Ltd., model 7170A, Japan) and Magnesium measurement kits (Hitachi Ltd., Japan). Analyzing wavelength=546 nm.
**Reasons for exclusion**	Study participants did not have either preeclampsia or eclampsia.

**Liu 1982**	**Characteristics**
**Study design**	Cross-sectional study
**Study objective**	To determine the safety of large $MgSO_4$ dose and routes of administration.
**Participants**	10 women with preeclampsia. The definition of preeclampsia was not stated same as the criteria for exclusion from the study.<br>Setting: Department of Obstetrics and Gynecology, Xiahua Hospital, Shanghai.
**Dosage regimen**	Different dosages of $MgSO_4$; IV 5 gram (single dose), IM 5 gram (single dose), 4-5 gram IV + IM 5-10 gram (single loading dose), 1 gram/hour continuous infusion.
**Outcomes**	Serum concentration and clinical observation.
**Laboratory method of estimating serum magnesium concentration**	Method used to measure serum magnesium level was not reported.
**Reasons for exclusion**	A single Concentration-Time Curve was used to present mean serum level of the different dosage regimens. This curve was not good enough to enable extraction of serum level at different time intervals for the regimens used.

**Lu 2000**	**Characteristics**
**Study design**	Non-systematic review of the literature on pharmacokinetic principles of magnesium sulphate in eclampsia.
**Study objective**	To outline the current available knowledge of the $MgSO_4$ and its clinical use.
**Participants**	Not applicable.
**Dosage regimen**	Not applicable
**Outcomes**	Not applicable
**Laboratory method of estimating serum magnesium concentration**	Not applicable
**Reasons for exclusion**	Non-systematic review of the literature.

**Pritchard 1955**	**Characteristics**
**Study design**	Cross-sectional study
**Study objective**	To demonstrate the pharmacokinetic and pharmacokinetic profile of MgSO4 when used for preeclampsia and eclampsia.
**Participants**	211 patients managed for severe preeclampsia and eclampsia classified as patients with moderately severe, severe preeclampsia, chronic hypertension with superimposed preeclampsia and eclampsia.<br>Exclusion criteria: Not stated<br>Setting: Department of Obstetrics and Gynecology, western Reserve University School of Medicine and University Hospitals of Cleveland, USA.
**Dosage regimen**	Eclampsia - 14 (4 gram IV + 10 gram IM) gram loading dose of MgSO4 followed by IM 5 gram 4 hourly maintenance dose<br>Preeclampsia - 10 gram IM loading dose followed by 5 gram IM 4 hourly maintenance dose of MgSO4.
**Outcomes**	Serum, cerebrospinal and urinary magnesium.
**Laboratory method of estimating serum magnesium concentration**	Either spectrophotometric method with titan yellow or by Briggs in which magnesium was precipitated as magnesium ammonium phosphate.
**Reasons for exclusion**	The study, though, had 211 participants over a 3-year period, reported its findings using variable number of participants' data to support assertions. For example, mean serum and CSF magnesium level reported were for both normal and preeclamptic pregnant women, spinal fluid magnesium level was reported for 1 woman after 7 days of MgSO4 treatment to be 3.5mEq/L while the average of CSF magnesium of 10 others was 2.65mEq/L.

**Pritchard 1980**	**Characteristics**
**Study design**	Non-systematic review of the literature on management of preeclampsia and eclampsia.
**Study objective**	
**Participants**	Not applicable
**Dosage regimen**	Not applicable
**Outcomes**	Not applicable
**Laboratory method of estimating serum magnesium concentration**	Not applicable
**Reasons for exclusion**	Non-systematic review

Rui 1996	Characteristics
**Study design**	Open label RCT study design
**Study objective**	To develop population PK and PKPD (blood pressure) models for MgSO4 in women with pregnancy induced hypertension.
**Participants**	60 participants with pregnancy induced hypertension (BP>= 20.3/13.3 kpa, Serum creatinine = 68-106 umol/L, Urine protein = ++ to ++++, no previous anti-convulsion therapy for at least a week). Most participants were young, mean age of 27.25 (SD=3.13) years and they had a mean weight of 68.36 (SD=10.06) Kg.<br>Exclusion criteria: Not stated<br>Setting: General Hospital of Nanjing Armed Forces, Nanjing, P.R. China.
**Dosage regimen**	Fast IV infusion for 1h followed by slow IV infusion for 5h (exact dose unreported).
**Outcomes**	Serum magnesium (mmol/L). No individual values; no associated time points post dose; only a mean/SD for all serum samplings from all participants.<br><br>Population 2 compartment PK model: K10, K12, K21 and Vc parameters estimates and associated between subjects variability.<br><br>Population Emax effect compartment PD model: Emax, C50 and Keo estimates and associated between subjects variability.
**Laboratory method of estimating serum magnesium concentration**	Serum total magnesium was estimated with an atomic absorption spectroscope (HP model 8452). Analyzing wavelength=540 nm, reference wavelength = 630-680 nm. Linear range=0.5-3.6 mmol/L. Precision RSD<2.6%. Recovery%=100.4%+/-1/2%.
**Reasons for exclusion**	No serum magnesium levels, be it baseline or post dose, was estimated and reported

Standley 1997	Characteristics
**Study design**	Cross sectional study
**Study objective**	To compare serum levels of ionized and total magnesium with those of calcium, sodium and potassium over the course of pregnancy.
**Participants**	31 normal primigravida (out of whom 9 women developed preeclampsia) were monitored in pregnancy for trend in serum magnesium in each trimester.<br>Exclusion criteria: Not stated<br>Setting: Hutzel Hospital, Detroit, Michigan, USA.
**Dosage regimen**	None
**Outcomes**	Serum magnesium, calcium, sodium and potassium.
**Laboratory method of estimating serum magnesium concentration**	Ionised magnesium was estimated using Nova 8 Biomedical, Inc., Waltham, MA) and total magnesium was estimated using Nova nucleus chemistry analyser.
**Reasons for exclusion**	None of those who developed preeclampsia had $MgSO_4$ and the study reported physiologic trend of serum magnesium in pregnancy.

Tudela 2012	Characteristics
**Study design**	Retrospective analysis of Hospital Database
**Study objective**	To estimate the impact of body mass index (BMI) on serum magnesium levels in women receiving infusions for eclampsia prophylaxis.
**Participants**	2141 women who had severe preeclampsia and had records of their body mass index (BMI).<br>Exclusion criteria: Not stated.<br>Setting: Department of Obstetrics and Gynecology, University of Texas Southwestern Medical center, Dallas, Texas, USA.
**Dosage regimen**	Intravenous 6 gram loading dose and 2 gram per hour maintenance dose of $MgSO_4$.
**Outcomes**	Serum magnesium.
**Laboratory method of estimating serum magnesium concentration**	Method used to measure serum magnesium was not reported.
**Reasons for exclusion**	Abstract of a study which formed part; last 3 years of Tudela 2013. In addition, study reported serum magnesium levels as “therapeutic” or “subtherapeutic” and made comparisons with weight categories. No individual participant’s serum level was presented.

Tudela 2013	Characteristics
**Study design**	Retrospective analysis of Hospital Database
**Study objective**	To estimate the effect of body mass index (BMI) on magnesium levels for eclampsia prophylaxis. Participants were categorized into under-weight, normal weight, over-weight and obese women.
**Participants**	5304 women who had severe preeclampsia and had records of their body mass index (BMI). Severe preeclampsia was defined as blood pressure ≥140/90 mmHg after 20 weeks of gestation in a woman without chronic hypertension, ≥2+ proteinuria by dipstick in a catheterized urine specimen, serum creatinine > 1.2mg/dl, platelets < 100,000, liver enzymes elevated two times the normal, persistent headache, and mid epigastric or upper quadrant pain.<br>Exclusion criteria: Not stated.<br>Setting: Department of Obstetrics and Gynecology, University of Texas Southwestern Medical center, Dallas, Texas, USA.
**Dosage regimen**	Intravenous 6 gram loading dose and 2 gram per hour maintenance dose of $MgSO_4$.
**Outcomes**	Serum magnesium relative to BMI.
**Laboratory method of estimating serum magnesium concentration**	Method used to measure serum magnesium was not reported.
**Reasons for exclusion**	Study reported serum magnesium levels as “therapeutic” or “subtherapeutic” and made comparisons with weight categories. No individual participant’s serum level was presented.
