# Supplementary material for: Clinical pharmacokinetic properties of magnesium sulphate in women with pre‐eclampsia and eclampsia
Source: BJOG. 2015 Nov 24;123(3):356–66. doi: 10.1111/1471-0528.13753 (PMC4737322; doi:10.1111/1471-0528.13753)
Supplement: Supplementary file 7 — Table S4. Risk of bias assessment of included studies. [file BJO-123-356-s007.docx]

**Supplementary table S4: Risk of bias assessment of included studies**

| **Study** | **Domain of assessment** | **Risk of bias**  **Authors’ judgment** | **Support for judgment** |
| --- | --- | --- | --- |
| **Aali et al.^8^** | Number of participants | Low | 50 participants; 46 women with severe preeclampsia and 4 women with eclampsia |
|  | Was the spectrum of patients representative of the patients who will receive the test in practice | Low | Severe preeclampsia was defined as blood pressure ≥160/110mmHg and proteinuria ≥2+. Eclampsia was taken as convulsion in a woman with preeclampsia not attributable to other causes |
|  | Did the study adequately report participants’ demographic characteristics (e.g. maternal age, gestation age, race, body weight / body mass index)? | Unclear | Maternal age, weight and gestation age were reported as mean± SD |
|  | Extent to which study objective align with the systematic review objective | Low | To determine the correlation between ionized and total magnesium under baseline and therapeutic conditions |
|  | Was laboratory method used to estimate serum magnesium described in detail? | Low | Serum total magnesium was estimated with an atomic absorption spectroscope (Shimadzu 620. Tokyo Japan) while ionized magnesium was analyzed by dye-binding colorimetry in which magnesium ion forms a red complex with Eriochrom black-T in alkaline solution (Shimadzu-2100). |
|  | Is the technology of index test unchanged since the study was carried out? | Low | Atomic absorption spectroscopy is still in use for estimation of serum magnesium |
|  | Baseline reporting and duration of post-dose estimation of serum magnesium | Low | MgSO_4_ was administered for 24 hours and serum magnesium was estimated up to 4 hours after stopping the regimen |
|  | Were withdrawals from the study explained | Unclear | It is impossible to say if results were provided for all women. |
| **Abbade et al.^12^** | No of participants sampled | Low | 29 women; 28 with preeclampsia and 1 woman with eclampsia. |
|  | Was the spectrum of patients representative of the patients who will receive the test in practice | Unclear | The definition of preeclampsia was not reported. Women with seizures that could be attributed to other causes in a woman with preeclampsia were excluded. |
|  | Did the study adequately report participants’ demographic characteristics (e.g. maternal age, gestation age, race, body weight / body mass index) ? | Unclear | Maternal age, weight and gestational age were reported as mean± SD |
|  | Extent to which study objective align with the systematic review objective | Low | To determine whether magnesium serum concentrations in patients with severe preeclampsia or eclampsia treated with two different regimens were different. |
|  | Was laboratory method used to estimate serum magnesium described in detail? | Unclear | “Magnesium plasma level was determined by Johnson & Johnson Vitros 950 dry-chemistry colorimetric analyzer. |
|  | Is the technology of index test unchanged since the study was carried out | Unclear | Unable to ascertain. |
|  | Baseline reporting and duration of post-dose estimation of serum magnesium | High | Baseline reported but duration of post-dose estimation of magnesium was 6 hours |
|  | Were withdrawals from the study explained | Low | Data were provided for 29 women. |
| **Apostol et al.^13^** | No of participants sampled | Low | 16 women with preeclampsia |
|  | Was the spectrum of patients representative of the patients who will receive the test in practice | Unclear | Preeclampsia was diagnosed by “classic criteria: hypertension, edema and proteinuria”. |
|  | Did the study adequately report participants’ demographic characteristics (e.g. maternal age, gestation age, race, body weight / body mass index)? | Unclear | Despite intrapartum administration of MgSO_4_, there was no information provided on gestational age and the weight of participants. Maternal age was presented as mean ± SEM. |
|  | Extent to which study objective align with the systematic review objective | Low | To correlate magnesium levels of serum with those of CSF in preeclamptic women receiving I.V MgSO_4_ in therapeutic doses and determine whether magnesium crosses the blood-brain barrier after I.V MgSO_4_. |
|  | Was laboratory method used to estimate serum magnesium described in detail? | High | Total Mg was measured by “standard techniques” |
|  | Is the technology of index test unchanged since the study was carried out | Unclear | Method used to estimate serum magnesium was not reported |
|  | Baseline reporting and duration of post-dose estimation of serum magnesium | Unclear | Intrapartum MgSO_4_ after which, blood was drawn every 6 hours”. |
|  | Were withdrawals from the study explained | Low | Results were provided for all women. |

| **Study** | **Domain of assessment** | **Risk of bias**  **Authors’ judgment** | **Support for judgment** |
| --- | --- | --- | --- |
| **Chen et al.^14^** | No of participants sampled | Low | 30 women. |
|  | Was the spectrum of patients representative of the patients who will receive the test in practice | Low | Pregnancy induced hypertension defined as (BP 140-190 mmHg/100-120 mmHg, Urine protein ++ to ++++, edema ++, with headache/vomit/nausea, note normal kidney function). |
|  | Did the study adequately report participants’ demographic characteristics (e.g. maternal age, gestation age, race, body weight / body mass index)? | Unclear | Age range of 24-36 years old. No other demographic characteristics were provided. |
|  | Extent to which study objective align with the systematic review objective | Low | To develop population pharmacokinetic models for MgSO4 in women with pregnancy induced hypertension. And to investigate the associated blood-pressure lowering effect. |
|  | Was laboratory method used to estimate serum magnesium described in detail? | Unclear | Magnesium levels were estimated using colorimetric end-points with the linear range, peculiarities of laboratory method reported. |
|  | Is the technology of index test unchanged since the study was carried out | Unclear | Unable to ascertain because of partial description of method. |
|  | Baseline reporting and duration of post-dose estimation of serum magnesium | Unclear | Serum magnesium estimation performed up to 15 hours after initiation of MgSO_4_ infusion |
|  | Were withdrawals from the study explained | Unclear | No explanation provided for withdrawals. |
| **Chesley and Tepper ^3^** | No of participants sampled | Low | 52 women with either preeclampsia or eclampsia |
|  | Was the spectrum of patients representative of the patients who will receive the test in practice | Unclear | Definition of preeclampsia not given and authors included cases of mild preeclampsia (about half of participants). |
|  | Did the study adequately report participants’ demographic characteristics (e.g. maternal age, gestation age, race, body weight / body mass index)? | High | No participants’ demographic characteristics were provided |
|  | Extent to which study objective align with the systematic review objective | Low | To know what plasma magnesium levels would follow various doses of magnesium sulfate, given in various ways. |
|  | Was laboratory method used to estimate serum magnesium described in detail? | Unclear | “Serum magnesium was measured by the method of Simonsen, Westover and Wertman using the Beckman DU spectrophotometer”. |
|  | Is the technology of index test unchanged since the study was carried out | Unclear | Unable to ascertain |
|  | Baseline reporting and duration of post-dose estimation of serum magnesium | Unclear | Baseline reporting was performed but post-dose estimation of serum magnesium was for 6 hours. |
|  | Were withdrawals from the study explained | Unclear | The number of women who had 10 g intramuscular (I.M) loading dose and a single 5 g IM maintenance dose was not stated. Therefore the total number of participants who had different regimens of MgSO_4_ did not add up to 52 as reported in the methods section of the study. |

| **Study** | **Domain of assessment** | **Risk of bias**  **Authors’ judgment** | **Support for judgment** |
| --- | --- | --- | --- |
| **Chesley ^15^** | Number of participants | High | Three participants with preeclampsia. Definition of preeclampsia was not provided. |
|  | Was the spectrum of patients’ representative of the patients who will receive the test in practice? | Unclear | The definition of preeclampsia was not provided. |
|  | Did the study adequately report participants’ demographic characteristics (e.g. maternal age, gestation age, race, body weight / body mass index)? | High | No participants’ demographic characteristics were provided |
|  | Extent to which study objective align with the systematic review objective | High | Study objective was to measure the apparent volume of distribution of Sucrose and Magnesium. |
|  | Was laboratory method used to estimate serum magnesium described in detail? | Unclear | “Serum magnesium was measured by the method of Simonsen” |
|  | Is the technology of index test unchanged since the study was carried out | Unclear | Unable to ascertain. |
|  | Baseline reporting and duration of post-dose estimation of serum magnesium | Unclear | Baseline reporting was not performed and serum magnesium estimation was stopped at 4 hours |
|  | Were withdrawals from the study explained | Low | Data was provided for the three women. |
| **Chissell et al.^16^** | No of participants sampled | Low | 17 women |
|  | Was the spectrum of patients representative of the patients who will receive the test in practice | Low | Severe preeclampsia and imminent eclampsia defined as proteinuria of at least 1+ on dipstick method (Ames) and diastolic blood pressure of ≥ 110mmHg. Imminent eclampsia was defined persistent headaches, visual disturbance, epigastric pain, increased patellar reflexes and clonus |
|  | Did the study adequately report participants’ demographic characteristics (e.g. maternal age, gestation age, race, body weight / body mass index) ? | Unclear | The age and weight of the women was reported as means. The gestational age at participation in the study was not reported. |
|  | Extent to which study objective align with the systematic review objective | Low | To compare the clinical outcome as well as the magnesium levels obtained in the treatment of severe preeclampsia with IM regimen of Pritchard and I.V regimen with maintenance doses of 2 g/h. |
|  | Was laboratory method used to estimate serum magnesium described in detail? | Unclear | “Samples analyzed the following day using dye-binding procedure with Calmagite (Beckman Sychron CX5)”. |
|  | Is the technology of index test unchanged since the study was carried out | Unclear | Unable to ascertain |
|  | Baseline reporting and duration of post-dose estimation of serum magnesium | Unclear | Sample collection was done at baseline and up to 19 hours after MgSO_4_ administration. |
|  | Were withdrawals from the study explained | Low | Data were provided for all women. |
| **Chuan et al.^17^** | No of participants sampled | Low | 116 women with severe preeclampsia |
|  | Was the spectrum of patients representative of the patients who will receive the test in practice | Low | Severe preeclampsia was defined as BP>160/110mmHg, persistent severe headaches, visual disturbances, more than 2 clonus, elevated aspartate or alanine transferase levels to twice normal with epigastric or right upper-quadrant pain |
|  | Did the study adequately report participants’ demographic characteristics (e.g. maternal age, gestation age, race, body weight / body mass index)? | Unclear | The race of participants was reported and the mean of their ages. However, the weight of the participants was not reported. |
|  | Extent to which study objective align with the systematic review objective | Low | To determine the population pharmacokinetics of magnesium from sparse observational data in patients with preeclampsia. |
|  | Was laboratory method used to estimate serum magnesium described in detail? | Low | Magnesium assay was determined by the dye-binding colorimetry in which magnesium ion formed a purple-red complex with xylidyl blue in alkaline solution and detected at 600nm and NONMEM program in a 1-compartment model. |
|  | Is the technology of index test unchanged since the study was carried out | Unclear | Use of dye-binding colorimetry and NONMEM pharmacokinetic model |
|  | Baseline reporting and duration of post-dose estimation of serum magnesium | Unclear | Baseline serum magnesium was reported. However, other results were from the model which did not include serum magnesium levels up to 24 hours post MgSO_4_ use. |
|  | Were withdrawals from the study explained | Low | Data on 116 women were provided. |

| **Study** | **Domain of assessment** | **Risk of bias**  **Authors’ judgment** | **Support for judgment** |
| --- | --- | --- | --- |
| **Cruikshank et al.^18^** | Number of participants | Low | 20 women |
|  | Was the spectrum of patients’ representative of the patients who will receive the test in practice?. | Unclear | Inclusion and exclusion criteria were not provided by the authors, neither was definition of mild or moderate preeclampsia made. |
|  | Did the study adequately report participants’ demographic characteristics (e.g. maternal age, gestation age, race, body weight / body mass index)? | High | The demographic characteristics of participants were not provided. |
|  | Extent to which study objective align with the systematic review objective | High | Study objective was to investigate the cause of magnesium-induced hypocalcaemia |
|  | Was laboratory method used to estimate serum magnesium described in detail? | Low | Urine samples were collected in aliquots from an in-dwelling catheter. Maternal blood was collected before and after the infusion. Magnesium determination in urine and blood were done by atomic absorption spectrophotometry (Perkin-Elmer model 303). |
|  | Is the technology of index test unchanged since the study was carried out | Low | Atomic absorption spectrophotometry is still in use |
|  | Baseline reporting and duration of post-dose estimation of serum magnesium | Low | Baseline and post –dose serum magnesium were estimated up to 24 hours |
|  | Were withdrawals from the study explained | Unclear | It is impossible to ascertain withdrawals from the study |
| **Dayicioglu et al.^19^** | No of participants sampled | Low | 194 women |
|  | Was the spectrum of patients representative of the patients who will receive the test in practice | Low | Hypertension was defined as blood pressure 140/90mm Hg, using Korotkoff phase V to define diastolic pressure. Proteinuria was described as 300 mg or more of proteinuria per 24 hours or persistent 30 mg/dl (1+dipstick) in random urine samples. Preeclampsia was considered severe in the presence of persistent high systolic (160mm Hg) and diastolic (110mm Hg) blood pressure despite antihypertensive therapy, persistent severe headache, visual disturbance, and elevated aspartate or alanine transferase levels with epigastric or right upper-quadrant pain. |
|  | Did the study adequately report participants’ demographic characteristics (e.g. maternal age, gestation age, race, body weight / body mass index)? | Unclear | Participants’ weight, age and gestational age were reported using the mean. |
|  | Extent to which study objective align with the systematic review objective | Low | To determine if with standard dose of MgSO4 to preeclamptic women there is a significant change in serum magnesium levels according to body mass indices and if the changes affect the rate of preventing eclamptic seizures. |
|  | Was laboratory method used to estimate serum magnesium described in detail? | Unclear | Laboratory method was unreported. |
|  | Is the technology of index test unchanged since the study was carried out | Unclear | Laboratory method was unreported. |
|  | Baseline reporting and duration of post-dose estimation of serum magnesium | Unclear | No baseline serum estimation was recorded. However, post dose serum magnesium was estimated up to 42 hours. |
|  | Were withdrawals from the study explained | Low | Results were provided for all women. |

| **Study** | **Domain of assessment** | **Risk of bias**  **Authors’ judgment** | **Support for judgment** |
| --- | --- | --- | --- |
| **Ekele and Badung ^20^** | Number of participants | Low | 19 Women with eclampsia. |
|  | Was the spectrum of patients’ representative of the patients who will receive the test in practice? | Unclear | The definition of eclampsia was not provided. |
|  | Did the study adequately report participants’ demographic characteristics (e.g. maternal age, gestation age, race, body weight / body mass index)? | Unclear | Participants’ body mass index and gestational age were reported using the mean. |
|  | Extent to which study objective align with the systematic review objective | Low | To estimate the serum levels of magnesium in eclamptic women on magnesium sulphate and relate serum levels with clinical findings |
|  | Was laboratory method used to estimate serum magnesium described in detail? | Unclear | Serum magnesium assayed using Xylidyl blue colorimetric principle and Jenway 6051 colorimeter. |
|  | Is the technology of index test unchanged since the study was carried out | Unclear | Unable to ascertain |
|  | Baseline reporting and duration of post-dose estimation of serum magnesium | Unclear | Baseline was reported. However, post-dose serum magnesium was estimated up to 12^th^ hour. |
|  | Were withdrawals from the study explained | Low | Results were provided for all women. |
| **Guzin et al.^21^** | No of participants sampled | Low | 50 women |
|  | Was the spectrum of patients representative of the patients who will receive the test in practice | Low | Severe preeclampsia was defined as blood pressure ≥160/110 mmHg on two occasions, 6 hours apart or, if proteinuria was 5 gram or more in 24 hours. Severity of preeclampsia was included features of end-organ damage and laboratory abnormalities of HELLP. Eclampsia was occurrence of convulsion in preeclamptic cases due to no other causes. |
|  | Did the study adequately report participants’ demographic characteristics (e.g. maternal age, gestation age, race, body weight / body mass index) ? | High | No report of participants’ age and weight was given. In addition, the table on demographic features was misinterpreted as “gestational age” and “gestational week” were presented. |
|  | Extent to which study objective align with the systematic review objective | High | The study objective was to assess the effect of magnesium sulfate on blood biochemical parameters and coagulation status. |
|  | Was laboratory method used to estimate serum magnesium described in detail? | High | The method used was not reported. |
|  | Is the technology of index test unchanged since the study was carried out | Unclear | The method used not reported |
|  | Baseline reporting and duration of post-dose estimation of serum magnesium | Unclear | Measured baseline serum magnesium and then 2 hour-post dose magnesium. |
|  | Were withdrawals from the study explained | Unclear | It is impossible to determine whether results were provided for all women. |
| **Handwerker et al.^22^** | No of participants sampled | Low | 8 women |
|  | Was the spectrum of patients representative of the patients who will receive the test in practice | Low | Preeclampsia defined by elevated blood pressure ≥140/90 mmHg and at least 1+ proteinuria by semi-quantitative assay. |
|  | Did the study adequately report participants’ demographic characteristics (e.g. maternal age, gestation age, race, body weight / body mass index)? | Unclear | No information on maternal age was provided and the age and gestational age of participants were reported using the mean. |
|  | Extent to which study objective align with the systematic review objective | Low | To demonstrate the levels of magnesium ion change during standard intravenous MgSO_4_ therapy. |
|  | Was laboratory method used to estimate serum magnesium described in detail? | Low | Ionized and total serum magnesium were analyzed with NOVA Biomedical Stat Profile 8 Analyzer and Kodak Ektachem DT-60 Analyzer respectively. Reference was made to complete description of method. |
|  | Is the technology of index test unchanged since the study was carried out | Unclear | The authors referred to details of procedure in one of the references, though the equipment use was specified in the study. |
|  | Baseline reporting and duration of post-dose estimation of serum magnesium | Unclear | Baseline reported but post-dose serum magnesium estimation stopped at 3 hours. |
|  | Were withdrawals from the study explained | Unclear | It is impossible to say if results were provided for all women. |

| **Study** | **Domain of assessment** | **Risk of bias**  **Authors’ judgment** | **Support for judgment** |
| --- | --- | --- | --- |
| **Lu et al.^23^** | Number of participants | Low | 51 women |
|  | Was the spectrum of patients’ representative of the patients who will receive the test in practice? | Low | Gestational hypertension was defined as systolic blood pressure of at least 140 mmHg with a >30mmHg rise and/or diastolic blood pressure of at least 90mmHg with a rise of >15nnHg occurring on two or more occasions after 20 weeks of gestation. Proteinuria was defined as a urine protein concentration of at least “2+”. |
|  | Did the study adequately report participants’ demographic characteristics (e.g. maternal age, gestation age, race, body weight / body mass index)? | High | No information on the demographic characteristics of participants was provided. |
|  | Extent to which study objective align with the systematic review objective | High | The study objective was to describe the relationship between plasma magnesium concentration and blood pressure response in pregnant women with preeclampsia |
|  | Was laboratory method used to estimate serum magnesium described in detail? | High | The method was not reported. |
|  | Is the technology of index test unchanged since the study was carried out | Unclear | Despite the unreported method of serum magnesium estimation, NONNEM program was used to get some data. |
|  | Baseline reporting and duration of post-dose estimation of serum magnesium | Unclear | Only baseline serum magnesium was reported, though in the methods, the authors planned to collect maternal blood up to 15 hours after starting MgSO_4_ infusion. |
|  | Were withdrawals from the study explained | Unclear | No data was provided on demographic characteristics of participants that could allow judgment to be made. |
| **Manorot et al.^24^** | No of participants sampled | Low | 50 women |
|  | Was the spectrum of patients representative of the patients who will receive the test in practice | Unclear | The diagnostic criteria for severe preeclampsia were not stated. |
|  | Did the study adequately report participants’ demographic characteristics (e.g. maternal age, gestation age, race, body weight / body mass index) ? | Unclear | Maternal age, weight and gestational age were reported as mean for the women. |
|  | Extent to which study objective align with the systematic review objective | Low | To compare serum level between intravenous and intramuscular maintenance regimen in women with severe preeclampsia |
|  | Was laboratory method used to estimate serum magnesium described in detail? | Unclear | Serum magnesium was determined by methylthymol blue method with the use of spectrophotometry |
|  | Is the technology of index test unchanged since the study was carried out | Unclear | Unable to ascertain |
|  | Baseline reporting and duration of post-dose estimation of serum magnesium | Unclear | No report of baseline magnesium and post-dose magnesium was estimated up to 6 hours. |
|  | Were withdrawals from the study explained | Unclear | It was impossible to determine if results were provided for all women. |
| **Mason et al.^25^** | No of participants sampled | Low | 37 Pregnant women |
|  | Was the spectrum of patients representative of the patients who will receive the test in practice | Unclear | No definition of preeclampsia was provided. |
|  | Did the study adequately report participants’ demographic characteristics (e.g. maternal age, gestation age, race, body weight / body mass index)? | High | No demographic characteristics of the women were provided. |
|  | Extent to which study objective align with the systematic review objective | High | The study objective was to correlate fetal ionized magnesium levels with maternal ionized magnesium during MgSO_4_ administration |
|  | Was laboratory method used to estimate serum magnesium described in detail? | Low | Maternal blood samples and fetal umbilical vein blood samples were collected anaerobically, immediately after delivery. Derived serum was stored at -70 degree Celsius. Ionized and total magnesium was analyzed with Nova Biomedical stat Profile 8 and Nova Nucleus Biomedical stat Profile analyzers respectively (Nova Biomedical, Waltham,Mass). |
|  | Is the technology of index test unchanged since the study was carried out | Unclear | Unable to ascertain |
|  | Baseline reporting and duration of post-dose estimation of serum magnesium | Unclear | No baseline serum magnesium was reported. Post-dose serum magnesium estimation was not up to 24 hours. |
|  | Were withdrawals from the study explained | Unclear | It was impossible to determine if results were provided for all women. |

| **Study** | **Domain of assessment** | **Risk of bias**  **Authors’ judgment** | **Support for judgment** |
| --- | --- | --- | --- |
| **Phuapradit et al.^26^** | Number of participants | Low | 44 women |
|  | Was the spectrum of patients’ representative of the patients who will receive the test in practice? | Unclear | The definition of severe preeclampsia was not provided. |
|  | Did the study adequately report participants’ demographic characteristics (e.g. maternal age, gestation age, race, body weight / body mass index)? | Unclear | Arithmetic mean of maternal age, weight and gestational age at recruitment were presented. |
|  | Extent to which study objective align with the systematic review objective | Low | To review the use of intravenous magnesium sulphate in preeclampsia. |
|  | Was laboratory method used to estimate serum magnesium described in detail? | Low | Serum magnesium was analysed by atomic absorption spectrophotometry either immediately or from frozen samples previously stored for up to 48 hours. |
|  | Is the technology of index test unchanged since the study was carried out | Low | Atomic absorption spectrophotometry is still in use |
|  | Baseline reporting and duration of post-dose estimation of serum magnesium | Unclear | No baseline serum magnesium estimation. Post-dose serum magnesium was estimated up to 24 hours |
|  | Were withdrawals from the study explained | Unclear | It is impossible to say if results were provided for all women. |
| **Salinger et al.^27^** | No of participants sampled | Low | 258 women |
|  | Was the spectrum of patients representative of the patients who will receive the test in practice | Unclear | The definition of preeclampsia used was not provided |
|  | Did the study adequately report participants’ demographic characteristics (e.g. maternal age, gestation age, race, body weight / body mass index) ? | Unclear | Arithmetic mean of maternal age, weight and gestational age at recruitment were presented. |
|  | Extent to which study objective align with the systematic review objective | Low | To compare magnesium sulphate concentrations achieved by intramuscular and intravenous regimens used for the prevention of eclampsia. |
|  | Was laboratory method used to estimate serum magnesium described in detail? | Unclear | Partial description. Two different equipment were used at the two study sites. |
|  | Is the technology of index test unchanged since the study was carried out | Unclear | Unable to ascertain. |
|  | Baseline reporting and duration of post-dose estimation of serum magnesium | Unclear | Baseline serum magnesium was report but estimation of serum magnesium ended at 12 hours. |
|  | Were withdrawals from the study explained | Low | Treatment interruption before blood sampling. Failure to collect blood sample and mislabeled blood sample. |
| **Seydoux et al.^28^** | No of participants sampled | Low | Five women |
|  | Was the spectrum of patients representative of the patients who will receive the test in practice | Low | Preeclampsia was defined as hypertension and proteinuria ≥300mg/24hr or >1 g/L in a random urine |
|  | Did the study adequately report participants’ demographic characteristics (e.g. maternal age, gestation age, race, body weight / body mass index)? | High | The women’s characteristics were not separated from those of women who did not receive MgSO_4_. |
|  | Extent to which study objective align with the systematic review objective | Low | To determine serum and lymphocyte magnesium concentrations during normal pregnancy and to compare the magnesium status in the third trimester of pregnancy between women with normal pregnancy, and those with gestational hypertension or pre-eclampsia |
|  | Was laboratory method used to estimate serum magnesium described in detail? | Unclear | Plasma magnesium was determined by atomic absorption spectrophotometry |
|  | Is the technology of index test unchanged since the study was carried out | Low | Atomic absorption spectrometry is still in use. |
|  | Baseline reporting and duration of post-dose estimation of serum magnesium | Unclear | Baseline serum magnesium was report but estimation of serum magnesium ended prior to 24 hours |
|  | Were withdrawals from the study explained | Low | Results were presented for the five women. |
| **Shreya et al.^29^** | No of participants sampled | Low | 27 women with eclampsia and 53 having imminent eclampsia |
|  | Was the spectrum of patients representative of the patients who will receive the test in practice | Low | Eclampsia was referred to convulsions in a woman with pre-eclampsia that cannot be attributed to other causes |
|  | Did the study adequately report participants’ demographic characteristics (e.g. maternal age, gestation age, race, body weight / body mass index)? | Unclear | Percentage of maternal age and gestational age at recruitment were presented |
|  | Extent to which study objective align with the systematic review objective | Low | To study efficacy of Single dose of MgSO_4_ and Pritchard regimen in treatment of imminent eclampsia and eclampsia and to correlate clinical and biochemical parameters of serum MgSO_4_ in both regimens |
|  | Was laboratory method used to estimate serum magnesium described in detail? | High | No description of method of serum magnesium estimation |
|  | Is the technology of index test unchanged since the study was carried out | Unclear | Unable to ascertain |
|  | Baseline reporting and duration of post-dose estimation of serum magnesium | Unclear | Baseline serum magnesium was report but estimation of serum magnesium ended at 4 hours |
|  | Were withdrawals from the study explained | Low | Results were presented for the 27 women with eclampsia. |

| **Study** | **Domain of assessment** | **Risk of bias**  **Authors’ judgment** | **Support for judgment** |
| --- | --- | --- | --- |
| **Sibai et al.^5^** | Number of participants | Low | 32 women |
|  | Was the spectrum of patients’ representative of the patients who will receive the test in practice? | Low | Mild preeclampsia was defined as blood pressure 140/90mm Hg with proteinuria. Severe preeclampsia was defined as blood pressure of at least 160/110mmHg (on two occasions, 6 hours apart) with proteinuria |
|  | Did the study adequately report participants’ demographic characteristics (e.g. maternal age, gestation age, race, body weight / body mass index)? | Low | The range of the women’s age, weight and gestational age was reported. |
|  | Extent to which study objective align with the systematic review objective | Low | To compare the magnesium levels obtained in the treatment of preeclamptic patients with the intramuscular regimen of Pritchard to the levels in the continuous intravenous regimens with maintenance doses of 1 gm/hr and 2 gm/hr. |
|  | Was laboratory method used to estimate serum magnesium described in detail? | Low | Atomic absorption spectrophotometry was used to estimate serum magnesium immediately or from frozen sample stored up to 48 hours after sample collection. |
|  | Is the technology of index test unchanged since the study was carried out | Low | Atomic absorption spectrophotometry is still in use. |
|  | Baseline reporting and duration of post-dose estimation of serum magnesium | Unclear | Baseline serum magnesium level was reported but post-dose serum magnesium estimation was not done up to 24 hours. |
|  | Were withdrawals from the study explained | Low | Results were provided for all women. |
| **Singh et al.^30^** | No of participants sampled | Low | 70 women |
|  | Was the spectrum of patients representative of the patients who will receive the test in practice | Low | Preeclampsia was defined as blood pressure 140/90mmHg or more, proteinuria and oedema with/without history of convulsions in the last trimester of pregnancy, during labour or within 48 hours of delivery |
|  | Did the study adequately report participants’ demographic characteristics (e.g. maternal age, gestation age, race, body weight / body mass index) ? | Unclear | The mean age of the women was reported. No information on their weight was provided and the reported gestational age included those of normal women. |
|  | Extent to which study objective align with the systematic review objective | Low | To estimate the serum magnesium levels in preeclampsia and eclampsia and to study the effect of using different regimens of magnesium sulphate. |
|  | Was laboratory method used to estimate serum magnesium described in detail? | Unclear | Atomic absorption spectrophotometer (Model AAS-4139) was used to measure the serum magnesium |
|  | Is the technology of index test unchanged since the study was carried out | Low | Atomic absorption spectrophotometry is still in use. |
|  | Baseline reporting and duration of post-dose estimation of serum magnesium | Low | Baseline serum estimation was done. Post-dose serum magnesium was estimated up to 32 hours after initiation of MgSO_4_. |
|  | Were withdrawals from the study explained | Unclear | It was impossible to determine if results were provided for all women. |
| **Suvarna et al.^31^** | No of participants sampled | Low | 126 women |
|  | Was the spectrum of patients representative of the patients who will receive the test in practice | High | The definition of preeclampsia and eclampsia were not provided. |
|  | Did the study adequately report participants’ demographic characteristics (e.g. maternal age, gestation age, race, body weight / body mass index)? | Unclear | The mean of the women’s age, weight and gestational age at recruitment was reported. |
|  | Extent to which study objective align with the systematic review objective | High | Not reported |
|  | Was laboratory method used to estimate serum magnesium described in detail? | High | Method of estimation of serum magnesium was not provided. |
|  | Is the technology of index test unchanged since the study was carried out | Unclear | Unable to ascertain. |
|  | Baseline reporting and duration of post-dose estimation of serum magnesium | High | Study did not report baseline or time-point serum magnesium levels. It reported only serum level at which respiratory depression and death occurred. |
|  | Were withdrawals from the study explained | Low | Results were provided for all women. |

| **Study** | **Domain of assessment** | **Risk of bias**  **Authors’ judgment** | **Support for judgment** |
| --- | --- | --- | --- |
| **Taber et al.^32^** | Number of participants | Low | Nine women |
|  | Was the spectrum of patients’ representative of the patients who will receive the test in practice? | Unclear | Definition of preeclampsia was not provided. |
|  | Did the study adequately report participants’ demographic characteristics (e.g. maternal age, gestation age, race, body weight / body mass index)? | Unclear | The range of the women’s age was reported. However, the body mass index and gestational age at recruitment were reported as means. |
|  | Extent to which study objective align with the systematic review objective | Low | To determine the correlation between ionized magnesium and total magnesium under basal and therapeutic conditions and to define the initiation and elimination pharmacokinetics of both forms during intravenous MgSO_4_ infusion |
|  | Was laboratory method used to estimate serum magnesium described in detail? | Low | Ionized magnesium was analyzed using ion-selective electrodes from whole blood with an ion-selective electrode (NOVA 8 Analyzer; NOVA Biomedical Corp, Waltham, Mass). Samples for total magnesium was prepared by centrifugation for 10 min at 2500 revolutions/min and serum magnesium analysis was done by a xylidyl blue spectrophotometric method from blood samples frozen at – 20 degree Celsius. |
|  | Is the technology of index test unchanged since the study was carried out | Unclear | Unable to ascertain. |
|  | Baseline reporting and duration of post-dose estimation of serum magnesium | Unclear | Baseline serum magnesium reported but sampling of post-dose serum magnesium was for 4 hours |
|  | Were withdrawals from the study explained | Low | Results were presented for all women. |
| **Tongsong et al. ^33^** | No of participants sampled | Low | 49 women, 24 in 1g/h regimen and 25 in 2g/h regimen |
|  | Was the spectrum of patients representative of the patients who will receive the test in practice | Unclear | Didn’t describe criteria for diagnosis |
|  | Did the study adequately report participants’ demographic characteristics (e.g. maternal age, gestation age, race, body weight / body mass index)? | Low | There were information on age, weight, BP and gestational age. |
|  | Extent to which study objective align with the systematic review objective | Low | The objectives of the study were to compare:   1. serum Mg level between 1g/h versus 2g/h regimen at 2 and 4 hours after loading dose and 2 hours after delivery 2. rate of achieving therapeutic level   between the 2 regimen |
|  | Was laboratory method used to estimate serum magnesium described in detail? | Unclear | Only describe that methylthymol method using spectrophotometer was used to measure serum Mg level |
|  | Is the technology of index test unchanged since the study was carried out | Low | Not changed throughout the study |
|  | Baseline reporting and duration of post-dose estimation of serum magnesium | High | Baseline serum Mg level was not reported and serum Mg level was measured up to only at 2 hours after delivery |
|  | Were withdrawals from the study explained | Low | No withdrawal. |
| **Thurnau et al.^34^** | No of participants sampled | Low | 10 women |
|  | Was the spectrum of patients’ representative of the patients who will receive the test in practice? | Low | Pritchard’s definition was referenced and used to recruit women. |
|  | Did the study adequately report participants’ demographic characteristics (e.g. maternal age, gestation age, race, body weight / body mass index)? | Unclear | It reported individual participant’s age and gestational age but did not report their weight. |
|  | Extent to which study objective align with the systematic review objective | High | Study objectives were to Correlate cerebrospinal fluid magnesium with magnesium level in the blood and to assess if magnesium crosses the blood brain barrier |
|  | Was laboratory method used to estimate serum magnesium described in detail? | Unclear | Assays of serum magnesium were done with automatic clinical analyzers (a modification of the methylthymol blue complexometric procedure). |
|  | Is the technology of index test unchanged since the study was carried out | Unclear | Details of description of method of magnesium assay were not provided. |
|  | Baseline reporting and duration of post-dose estimation of serum magnesium | High | Only a single blood sample was taken at the same time as when lumbar puncture was made. |
|  | Were withdrawals from the study explained | Low | Each woman had her results presented. |

| **Study** | **Domain of assessment** | **Risk of bias**  **Authors’ judgment** | **Support for judgment** |
| --- | --- | --- | --- |
| **Wright et al.^35^** | Number of participants | Low | 25 consecutive women |
|  | Was the spectrum of patients’ representative of the patients who will receive the test in practice? | Low | Preeclampsia was defined as blood pressure >140/90mmHg and 1+ proteinuria in catheterized urine specimen |
|  | Did the study adequately report participants’ demographic characteristics (e.g. maternal age, gestation age, race, body weight / body mass index)? | Unclear | The age of the women was not reported though their weight and gestational age were reported as means. |
|  | Extent to which study objective align with the systematic review objective | Low | To measure the apparent volume of distribution (AVOD) for magnesium (Mg) in preeclampsia and determine if a standard 4 gram loading dose of MgSO_4_ will attain therapeutic levels |
|  | Was laboratory method used to estimate serum magnesium described in detail? | Unclear | Magnesium levels were determined using Calmagite reaction and a Paramax System |
|  | Is the technology of index test unchanged since the study was carried out | Unclear | Unable to ascertain. |
|  | Baseline reporting and duration of post-dose estimation of serum magnesium | High | Sample of blood was collected two times; before MgSO_4_ use and 1 minute after MgSO_4_ administration |
|  | Were withdrawals from the study explained | Low | Results of all the women were provided. |
| **Yoshida et al.^36^** | No of participants sampled | Low | 16 preeclampsia women |
|  | Was the spectrum of patients representative of the patients who will receive the test in practice | Low | Preeclampsia was defined as blood pressure was ≥140/90 mmHg plus proteinuria or edema that is generalized and overt or both. |
|  | Did the study adequately report participants’ demographic characteristics (e.g. maternal age, gestation age, race, body weight / body mass index) ? | Unclear | Only the mean gestational age of the women was reported. |
|  | Extent to which study objective align with the systematic review objective | Low | To estimate the relationship between ionized and total Mg levels during MgSO4 administration in women with preterm labour and preeclampsia. |
|  | Was laboratory method used to estimate serum magnesium described in detail? | Unclear | Selective ion electrode (NOVA 8 Analyzer, NOVA Biomedical Corp, Waltham, MA)was used to measure ionized magnesium at bedside while total serum magnesium was measured in the laboratory with a Hitachi 7700 automatic analyzer |
|  | Is the technology of index test unchanged since the study was carried out | Unclear | Details of description of method of magnesium assay were not provided. |
|  | Baseline reporting and duration of post-dose estimation of serum magnesium | Unclear | Sample collection was not up to 24 hours post MgSO_4_ dose. |
|  | Were withdrawals from the study explained | Unclear | It is impossible to say if results were provided for all women. |
