# Supplementary material for: Clinical pharmacokinetic properties of magnesium sulphate in women with pre‐eclampsia and eclampsia
Source: BJOG. 2015 Nov 24;123(3):356–66. doi: 10.1111/1471-0528.13753 (PMC4737322; doi:10.1111/1471-0528.13753)
Supplement: Supplementary file 8 — Box S1. References to excluded studies. [file BJO-123-356-s008.pdf]

Box S1. References to excluded studies

- Aali S, Khazaeli P, Ghasemi F, Mehdizadeh A. Serum magnesium and calcium ions in patients with severe pre-eclampsia/eclampsia undergoing magnesium sulfate therapy. Med Sci Monit. 2007; 13 (4): CR191-4.
- Apostol A., et al. "Profile of cerebral spinal fluid and serum magnesium calcium sodium and potassium levels in pre-eclamptic women during administration of magnesium sulfate." European Journal of Anesthesiology 2009; 26: 156.
- Brookfield K, Su F, Drover D, Adelus M, Lyell D, Carvalho B. Pharmacokinetics of magnesium sulfate in pregnant women. American Journal of Obstetrics and Gynecology 2015; 212: S102
- Boriboohirunsarn D, Lertbunnaphong T, Suwanwanich M. Correlation of magnesium levels in cord blood and maternal serum among pre-eclamptic pregnant women treated with magnesium sulfate. J Obstet Gynaecol Res. 2012; 38(1): 247-52
- Chao A. The patellar reflex in preeclamptic women with subtherapeutic and therapeutic serum magnesium levels. J Reprod Med. 1990; 35(7): 678-81.
- Charoenvidhya D, Manotaya S. Magnesium sulfate maintenance infusion in women with preeclampsia: a randomized comparison between 2 gram per hour and 1 gram per hour. J Med Assoc Thai. 2013; 96(4): 395-8.
- Chen G, Lu JF, Cao W. [Pharmacokinetic-pharmacodynamic model of magnesium sulfate in treatment of pregnancy-induced hypertension]. Zhongguo Yao Li Xue Bao. 1991; 12(3): 222-5.
- Cruikshank DP, Varner MW, Pitkin RM. Breast milk magnesium and calcium

concentrations following magnesium sulfate treatment. Am J Obstet Gynecol. 1982; 143(6): 685-8.

- Fong J, Gurewitsch ED, Volpe L, Wagner WE, Gomillion MC, August P. Baseline serum and cerebrospinal fluid magnesium levels in normal pregnancy and preeclampsia. Obstet Gynecol. 1995; 85(3): 444-8
- Flowers CE Jr, Easterling WE Jr, White FD, Jung JM, Fox JT Jr. Magnesium sulfate in toxemia of pregnancy. New dosage schedule based on body weight. Obstet Gynecol. 1962; 19: 315-27.
- Flowers CE Jr. Magnesium sulfate in obstetrics. A study of magnesium in plasma, urine, and muscle. Am J Obstet Gynecol. 1965; 91:763-76.
- Ghahiri, A, Berjis K. "A comparison between intravenous magnesium sulfate and oral magnesium chloride in mild preeclampsia." Journal of Research in Medical Sciences 2005; 10(1): 6-9.
- Hallak M, Berry SM, Madincea F, Romero R, Evans MI, Cotton DB. Fetal serum and amniotic fluid magnesium concentration with maternal treatment. Obstet Gynecol 1993; 81:185-8.
- Huang Y, Zhang W, Wang L. [Study of magnesium and calcium levels of plasma and within erythrocyte before and after magnesium sulfate treatment in patients with pregnancy induced hypertension]. Zhonghua Fu Chan Ke Za Zhi. 1998; 33(6): 325-7.
- Liu DL, Pan ZR, Huang ZL. Different doses and routes of magnesium sulfate administration in treating severe preeclamptic and eclamptic patients. Chin Med J (Engl). 1982; 95(5): 363-6
- Lu JF, Nightingale CH. Magnesium sulfate in eclampsia and pre-eclampsia: pharmacokinetic principles. Clin Pharmacokinet. 2000; 38(4): 305-14.

- Pritchard JA. The use of Magnesium ion in the management of eclamptogenic toxaemia. Obstet Gynecol 1955; 100(2): 131-140.
- Pritchard JA. Management of preeclampsia and eclampsia. Kidney International, Vol. 18 (1980), pp. 259—266.
- Rui JZ, Yang YC, Wang YN, Chen G. [Population pharmacokinetics/pharmacodynamics of magnesium sulfate in pregnancy induced hypertensive gravidas treated with the drug]. Yao Xue Xue Bao. 1996; 31(2):81-5.
- Standley CA, Whitty JE, Mason BA, Cotton DB. Serum ionized magnesium levels in normal and preeclamptic gestation. Obstet Gynecol 1997; 89:24-7.
- Tudela, C., et al. (2012). "The impact of body mass index on serum magnesium sulfate levels in women receiving seizure prophylaxis." American Journal of Obstetrics and Gynecology 206(1): S356
- Tudela CM, McIntire DD, Alexander JM. Effect of maternal body mass index on serum magnesium levels given for seizure prophylaxis. Obstet Gynecol. 2013; 121(2 Pt): 314-20.
